# Antioxidant and Cytoprotective effects of *Pyrola decorata* H. Andres and its five phenolic components

**DOI:** 10.1186/s12906-019-2698-y

**Published:** 2019-10-21

**Authors:** Ban Chen, Xican Li, Jie Liu, Wei Qin, Minshi Liang, Qianru Liu, Dongfeng Chen

**Affiliations:** 1 School of Chinese Herbal Medicine, Guangzhou University of Chinese Medicine, Waihuan East Road No.232, Guangzhou Higher Education Mega Center, Guangzhou, 510006 China; 2 Innovative Research & Development Laboratory of TCM, Guangzhou University of Chinese Medicine, Guangzhou, 510006 China; 3 School of Basic Medical Science, Guangzhou University of Chinese Medicine, Guangzhou, 510006 China; 4 The Research Center of Integrative Medicine, Guangzhou University of Chinese Medicine, Guangzhou, 510006 China

**Keywords:** *Pyrola decorata*, *Luxiancao*, Mesenchymal stem cells, Hyperoside, 2′′-*O*-galloylhyperin, Cytoprotection, Antioxidant

## Abstract

**Background:**

*Pyrola decorata* H. Andres, is exclusively distributed in China and a source of traditional Chinese herbal medicine *Luxiancao* for more than 2000 years*.* Here, we evaluated the antioxidant and cytoprotective effects of *P. decorata* and its five phenolic components (protocatechuic acid, gallic acid, hyperoside, 2′′-*O*-galloylhyperin, and quercetin), and discussed their antioxidant chemistry.

**Methods:**

A lyophilized aqueous extract of *P. decorata* (**LAEP**) was prepared and analyzed with high-performance liquid chromatography (HPLC). **LAEP** and its five phenolic components were comparatively investigated using five antioxidant assays, including ferric-reducing antioxidant power, cupric ion-reducing antioxidant capacity, 2-phenyl-4,4,5,5-tetramethylimidazoline-1-oxyl-3-oxide radical (PTIO^•^)-scavenging, 1,1-diphenyl-2-picryl-hydrazl radical (DPPH^•^)-scavenging, and 2,2′-azino-bis(3-ethylbenzo-thiazoline-6-sulfonic acid) radical (ABTS^+•^)-scavenging activities. The reaction products of the five phenolic components with 4-methoxy-2,2,6,6-tetramethylpiperidine-1-oxyl radical (4-methoxy-TEMPO^•^) were determined with ultra-performance liquid chromatography coupled with electrospray ionization quadrupole time-of-flight tandem mass spectrometry (UPLC-ESI-Q-TOF-MS/MS) analysis. **LAEP** and its five phenolic components were incubated with bone marrow-derived mesenchymal stem cells (bmMSCs) subjected to oxidative stress to demonstrate their cytoprotective effects with a flow cytometry assay.

**Results:**

In the five antioxidant assays, **LAEP** and its five phenolic components dose-dependently increased the radical-scavenging (or reducing power) activities. However, the IC_50_ values of hyperoside were consistently higher than those of 2′′-*O*-galloylhyperin. UPLC-ESI-Q-TOF-MS/MS analysis results indicated that the five phenolics could yield dimer products in the presence of 4-methoxy-TEMPO^•^ via the radical adduct formation (RAF) pathway. Flow cytometry assay results confirmed the cytoprotective activity of **LAEP** and its five phenolic components toward stressed bmMSCs. In particular, 2′′-*O*-galloylhyperin could more effectively reduce the percentage of damaged bmMSCs than hyperoside.

**Conclusion:**

**LAEP** and its five phenolic components may undergo redox-based pathways (such as electron transfer and H^+^ transfer) and covalent-based pathway (i.e., RAF) to exhibit antioxidant activity. One consequence of RAF is the generation of phenolic-phenolic dimer. In both organic and aqueous media, 2′′-*O*-galloylhyperin exhibited higher redox-based antioxidant levels (or cytoprotective levels) than those with hyperoside. The differences could be attributed to 2′′-*O*-galloylation reaction.

## Background

*Pyrola* L. family comprises about 30 plant species, including *P. japonica* [1], *P. incarnata* Fisch [2], *P. decorata* H. Andres [3], and *P. renifolia* Maxim [4]. These plants are mainly distributed in temperate and cold temperate zones of the northern hemisphere [5]. *P. decorata*, however, is exclusively distributed in China [6].

The dried whole herb of *P. decorata* can act as the resource of Chinese herbal medicine *Luxianao* [7]. *Luxianao* however has been used in traditional Chinese medicine (TCM) for more than 2000 years. Modern pharmacological investigation has suggested that *Luxiancao* (or its bioactive components) exhibit anti-osteoarthritis [8], anti-inflammatory [8], hepatoprotective [9], and anti-influenza [10] properties. These pharmacological effects are known to be closely associated with its antioxidant and cytoprotective actions [4, 11–13]. However, the antioxidant mechanisms and cytoprotective effects of *Luxiancao* (*P. decorata*) have not been reported yet, regardless that its extract has been evaluated for the antioxidant capacity using chemical approach [4].

**Fig. 1:**
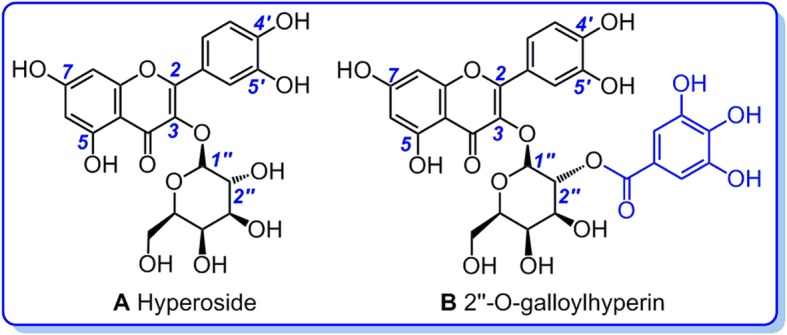
Structural formulae of hyperoside (**a**) and 2′′-O-galloylhyperin (**b**) (hyperoside is also called as hyperin)

To investigate the antioxidant mechanisms, the study tried to firstly analyze the antioxidant phenolics of *P. decorata* using HPLC technology. These phenolics were further measured for the radical adduct formation (RAF) possibility using ultra-performance liquid chromatography coupled with electrospray ionization quadrupole time-of-flight tandem mass spectrometry (UPLC-ESI-Q-TOF-MS/MS) technology. Especially, two flavonoids hyperoside (i.e., hyperin) and 2′′-*O*-galloylhyperin were comparatively analyzed in the study, this is because that: (1) they have been demonstrated to co-exist in *P. decorata* [4, 14, 15]; (2) Essentially, 2′′-*O*-galloylhyperin is a galloylation ester of hyperoside at 2′′-*O*-position (Fig. 1). Thereby, the comparison between them can be used to analyze the antioxidant structure-activity relationship of 2″-O-galloylation modification.

To explore the cytoprotective effects of *P. decorata*, bone marrow-derived mesenchymal stem cells (bmMSCs) were isolated from bone marrow of femur and tibia of rats [16, 17]. bmMSCs are known to possess characteristics of stem cells, including self-renewal and multi-directional differentiation properties [18], and bmMSC transplantation has been applied to treat heart diseases [19], spinal cord injuries [20] and diabetes [21]. During the process of diffusion and differentiation, chemical or physical factors, such as radiation and iron overload, could produce reactive oxygen species (ROS), resulting in the oxidative stress-induced damage to bmMSCs [22, 23]. The oxidative damage however has limited bmMSCs in transplantation engineering application. The study tried to use flow cytometric assay to evaluate the possible cytoprotective effect of *P. decorata* on oxidative stressed bmMSCs.

Apparently, the evaluation of the cytoprotective effects of this plant may improve our understanding of the beneficial properties of *Luxiancao*, and provide important information on its phenolics in MSCs transplantation engineering. The conclusion of the antioxidant structure-activity relationship of hyperoside and 2′′-*O*-galloylhyperin can also be used to explain the antioxidant difference between myricetin-3-*O*-rhamnopyranoside and myricetin-3-(2′′-*O*-galloyl)-*O*-rhamnopyranoside [24].

## Methods

### Reagents and materials

Protocatechuic acid (C_7_H_6_O_4_, CAS NO. 99–50-3, M.W. 154.1, purity 99%, Additional file 1), gallic acid (C_7_H_6_O_5_, CAS NO. 149–91-7, M.W. 170.1, purity 99%, Additional file 2), hyperoside (C_21_H_20_O_12_, CAS NO. 482–36-0, M.W. 464.4, purity 99%, Additional file 3), 2′′-*O*-galloylhyperin (C_28_H_24_O_16_, CAS NO. 53209–27-1, M.W. 616.5, purity 99%, Additional file 4), and quercetin (C_15_H_10_O_7_, CAS NO. 117–39-5, M.W. 302.2, purity 99%, Additional file 5) were obtained from Chengdu Biopurify Phytochemicals Ltd. (Chengdu, China). The compounds 2,9-dimethyl-1,10-phenanthroline (neocuproine), 1,1-diphenyl-2-picryl-hydrazl radical (DPPH^•^), ± − 6-hydroxyl-2,5,7,8-tetramethlychromane-2-carboxylic acid (Trolox), and 2,4,6-tripyridyl triazine (TPTZ) were purchased from Sigma-Aldrich Shanghai Trading Co., Ltd. (Shanghai, China), while 2-phenyl-4,4,5,5-tetramethylimidazoline-1-oxyl-3-oxide radical (PTIO^•^) was supplied by TCI Chemical Co. Ltd. (Shanghai, China). We purchased 2,2′-azino-bis(3-ethylbenzo-thiazoline-6-sulfonic acid) diammonium salt ([NH_4_]_2_ABTS) from Amresco Chemical Co. Ltd. (Solon, OH, USA), and 4-methoxy-2,2,6,6-tetramethylpiperidine-1-oxyl radical (4-methoxy-TEMPO^•^) from Tokyo Chemical Industry Co., Ltd. (Tokyo, Japan). Dulbecco’s modified Eagle’s medium (DMEM), trypsin, and fetal bovine serum (FBS) were procured from Gibco (Grand Island, NY, USA) and CD44 was supplied by Wuhan Boster Co., Ltd. (Wuhan, China). Annexin V/propidium iodide (PI) assay kit was obtained from Abcam (Cambridge, UK), and acetonitrile, from Merck Serono Co., Ltd. (Shanghai, China). Methanol, acetonitrile, high-performance liquid chromatography (HPLC)-grade water, and all other chemicals used were of analytical grade.

### Preparation of lyophilized aqueous extract of *P. decorata* (LAEP)

**Fig. 2:**
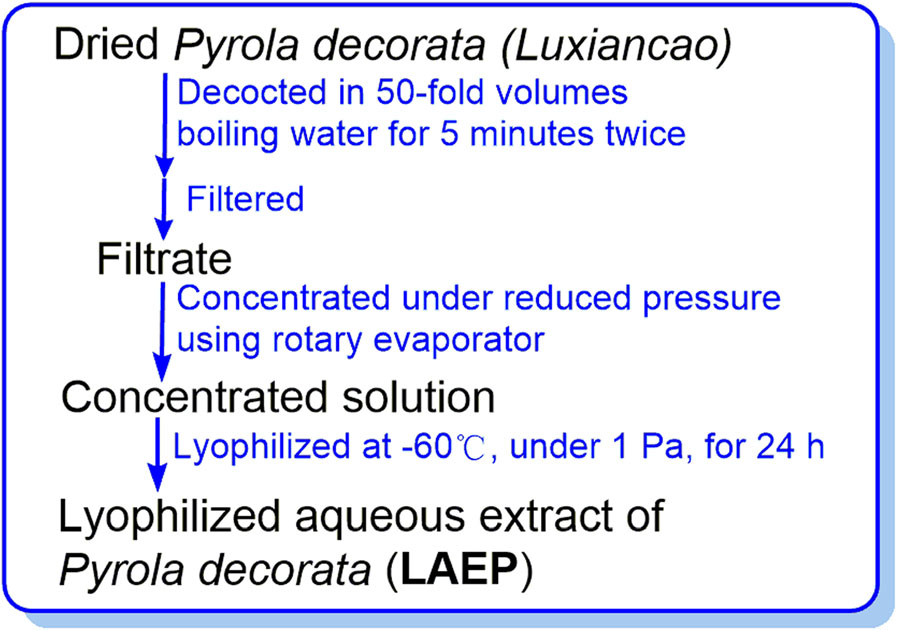
Preparation of **LAEP**

Dried *P. decorata* (*Luxiancao*) was purchased from Guangzhou Caizhilin Pharmaceutical Co., Ltd. (Guangzhou, China). It was identified by Dr. Guangtian Peng in School of Pharmaceutical Sciences, Guangzhou University of Chinese Medicine. A voucher specimen (No. GUCM010060) was deposited at Medicinal Plant Herbarium of Guangzhou University of Chinese Medicine (Additional file 6). *P. decorata* was subjected to extraction based on the guidance of the manufacturer (Fig. 2). In brief, the newly purchased *P. decorata* was decocted twice using 50-fold distilled water at 100 °C for 5 min. The extract was concentrated then lyophilized at − 60 °C under vacuum (1 Pa) for 24 h to obtain a lyophilized aqueous extract of *P. decorata* (**LAEP**). **LAEP** was stored at 5 °C until further use.

### HPLC analysis for phenolic components in LAEP

The **LAEP** powder was dissolved in methnol at 15 mg/mL and then filtered through a 0.45 μm nylon membrane. The filtrate was analyzed using HPLC on Shimadzu LC-20A (Shimadzu Co., Kyoto, Japan) equipped with Agilent 5 TC-C_18_ (250 mm × 4.6 mm, 5 μm) (Agilent Technologies Inc., Palo Alto, CA, USA). The mobile phase comprised A (0.1% formic acid in water) and B (acetonitrile) with a gradient elution as follows: 0–10 min (10–20% B), 10–20 min (20–30% B), and 20–30 min (30–50% B). The flow rate, injection volume, and column temperature were 1.0 mL/min, 10 μL, and 35 °C, respectively, and absorption was measured at 280 nm wavelength. Protocatechuic acid, gallic acid, hyperoside, 2′′-*O*-galloylhyperin, and quercetin were identified by comparing their retention times with those of authentic samples.

### Metal-reducing assays

The metal-reducing assays included ferric-reducing antioxidant power (FRAP) and cupric ion (Cu^2+^)-reducing antioxidant capacity (CUPRAC). The FRAP assay was carried out as per the method of Benzie and Strain [25]. In brief, the FRAP reagent was freshly prepared by mixing 10 mM TPTZ, 20 mM FeCl_3_, and 0.25 M acetate buffer (pH 3.6) at a ratio of 1:1:10. **LAEP** (5 mg/mL, *x* = 2–10 μL) or its five phenolic components (0.25 mg/mL, *x* = 2–10 μL) were added to (20 − *x*) μL of methanol and treated with 80 μL of FRAP reagent. After incubation for 30 min, the absorbance of the mixture was measured at 593 nm wavelength (A_593nm_) using acetate buffer as the blank on a microplate reader (Multiskan FC, Thermo Scientific, Shanghai, China). The relative reducing antioxidant power of the sample as compared to the maximum absorbance was calculated by the following formula:

$$ \mathrm{Relative}\ \mathrm{reducing}\ \mathrm{power}\%=\frac{\mathrm{A}-{\mathrm{A}}_{\mathrm{min}}}{{\mathrm{A}}_{\mathrm{max}}-{\mathrm{A}}_{\mathrm{min}}}\times 100\% $$

Where A_min_ is the lowest A_593nm_ value in the experiment, A is the A_593nm_ value of the reaction mixture with sample, and A_max_ is the greatest A_593nm_ value in the experiment.

The CUPRAC assay was conducted as per the method of Apak [26]. In brief, 12 μL CuSO_4_ aqueous solution (10 mM), 12 μL neocuproine methanol solution (7.5 mM), and (75 − *x*) μL CH_3_COONH_4_ buffer solution (100 mM, pH 7.4) were added to test tubes and incubated with different volumes of samples (10 mg/mL for **LAEP** or 0.5 mg/mL for the five phenolic components, *x* = 2–10 μL). The total volume was adjusted to 100 μL with buffer and the reactants were vigorously mixed. Absorbance against a buffer blank was measured at 450 nm wavelength using the microplate reader after 30 min. The relative reducing power of the sample as compared with the maximum absorbance was calculated using the formula presented for FRAP assay, wherein A_min_ is the lowest A_450nm_ value in the experiment, A is the A_450nm_ value of the reaction mixture with sample, and A_max_ is the greatest A_450nm_ value in the experiment.

### Free radical-scavenging assays in vitro

We performed three different free radical-scavenging assays, including 2-phenyl-4,4,5,5-tetramethylimidazoline-1-oxyl-3-oxide radical (PTIO^•^)-scavenging, 2,2′-azino-bis(3-ethylbenzo-thiazoline-6-sulfonic acid) radical (ABTS^+•^)-scavenging, and 1,1-diphenyl-2-picryl-hydrazl radical (DPPH^•^)-scavenging assays. The PTIO^•^-scavenging assay was conducted based on our previously published method [27]. The experimental procedures are briefly described as follows: PTIO^•^ radical was dissolved in phosphate buffers (0.1 mM, pH 4.5 and 7.4) to prepare a PTIO^•^ solution; **LAEP** (1 mg/mL at both pH 4.5 and 7.4) and its five phenolic components (0.5 mg/mL at pH 4.5 or 0.3 mg/mL at pH 7.4) were prepared using methanol. Various volumes (*x* = 2–10 μL) of samples were mixed with phosphate buffers at pH 4.5 and 7.4 and treated with PTIO^•^ solution (80 − *x* μL). After incubation for 3 h, the product mixture was analyzed by measuring the absorbance at 560 nm on microplate reader against a buffer blank. The PTIO^•^ inhibition percentage was calculated as follows:

$$ \mathrm{Inhibition}\%=\frac{{\mathrm{A}}_0-\mathrm{A}}{{\mathrm{A}}_0}\times 100\% $$

Where A_0_ is the absorbance at 560 nm of the control without the sample, and A is the absorbance of the reaction mixture with the sample. The above experiment was repeated using phosphate buffers at different pH (including pH 4.5 and 7.4).

The ABTS^+•^-scavenging activity was evaluated according to the previous method [28]. ABTS^+•^ was produced by mixing 0.2 mL of ABTS diammonium salt (7.4 mmol/L) with 0.2 mL of potassium persulfate (2.6 mmol/L). The mixture was incubated in the dark at room temperature (27 °C) for 12 h to complete the radical generation process before dilution with distilled water (at a ratio of approximately 1:20); the absorbance at 734 nm was 0.35 ± 0.01. To determine the scavenging activity, the *x* μL sample (*x* = 2–10 μL, 1 mg/mL for **LAEP** or 0.05 mg/mL for the five phenolic components) was added to (20 − *x*) μL of distilled water and treated with 80 μL of ABTS^+•^ reagent. The absorbance at 734 nm was measured 3 min after initial mixing using distilled water as the blank. The percentage of ABTS^+•^-scavenging activity was calculated using the formula presented for PTIO^•^-scavenging assay, wherein A_0_ is the absorbance at 734 nm of the control (reaction system without sample) and A is the absorbance at 734 nm of the reaction mixture with the sample.

The DPPH^•^ radical-scavenging activity was determined as previously described [29]. Briefly, 80 μL of DPPH^•^-methanolic solution (0.1 mol/L) was mixed with *x* μL sample-methanolic solution (for **LAEP:** *x* = 1–5 μL, the concentration was 2.5 mg/mL; for the five phenolics: *x* = 4–20 μL, the concentration was 0.01 mg/mL) and (20 – *x*) μL methanol. The mixture was maintained at room temperature for 30 min, and the absorbance was measured at 519 nm on microplate reader against a buffer blank. The percentage of DPPH^•^-scavenging activity was calculated based on the formula presented for PTIO^•^-scavenging assay, wherein A_0_ is the absorbance at 519 nm of the reaction system without sample and A is the absorbance at 519 nm of the reaction mixture with sample.

### Ultra-performance liquid chromatography coupled with electrospray ionization quadrupole time-of-flight tandem mass spectrometry (UPLC−ESI − Q − TOF − MS/MS) analysis

UPLC−ESI − Q − TOF − MS/MS analysis of reaction products of 4-methoxy-TEMPO^•^ with the five phenolic components was conducted as per the method described in our previous report [30]. The methanolic solutions of phenolic components were mixed with a solution of 4-methoxy-TEMPO^•^ in methanol at a molar ratio of 1:2, and the resulting mixtures were incubated for 24 h at room temperature. The product mixtures were filtered through a 0.22-μm filter and analyzed with a UPLC-ESI-Q-TOF-MS/MS system equipped with a C_18_ column (2.0 mm i.d. × 100 mm, 2.2 μm, Shimadzu Co., Kyoto, Japan). The mobile phase used for elution comprised a mixture of acetonitrile (phase A) and 0.1% formic acid in water (phase B). The column was eluted at a flow rate of 0.2 mL/min with the following gradient elution program: 0–2 min, maintained at 30% B; 2–10 min, 30–0% B; and 10–12 min, 0–30% B. The sample injection volume was set at 5 μL, while the column temperature was 35 °C.

The Q-TOF-MS/MS analysis was conducted on a Triple TOF 5600^*plus*^ mass spectrometer (AB SCIEX, Framingham, MA, USA) equipped with an ESI source in the negative ionization mode. The scan range was set at 100–2000 Da. The system was run with the following parameters: ion spray voltage, − 4500 V; ion source heater, 550 °C; curtain gas (CUR, N_2_), 30 psi; nebulizing gas (GS1, air), 50 psi; Tis gas (GS2, air), 50 psi. The declustering potential (DP) was set at − 100 V, whereas the collision energy (CE) was set at − 40 V at a collision energy spread (CES) of 20 V. The RAF products were quantified by extracting corresponding formula (e.g., [C_14_H_10_O_8_-H]^−^ for protocatechuic acid-protocatechuic acid dimer and [C_14_H_10_O_10_-H]^−^ for gallic acid-gallic acid dimer) from the total ion chromatogram and integrating the corresponding peak using PeakView 2.0 software (AB Sciex, Framingham, MA, USA).

### Protective effect on bmMSCs against ^•^OH-induced oxidative stress (flow cytometric assay)

Eight male Sprague-Dawley rats (4 weeks old, 60-80 g) were obtained from the Experimental Animal Center of Guangzhou University of Chinese Medicine (Guangzhou, China) and housed in there at 25 ± 2 °C, and exposed to a 12 h/12 h light–dark cycle with free access to food and water. Then, four Sprague-Dawley mice were euthanized by cervical dislocation to obtain enough bone marrow-derived mesenchymal stem cells (bmMSCs) from the bone marrow of femur and tibia of the rats.

The bmMSCs were cultured as previously described with some modifications [31]. Briefly, the cells were collected by gradient centrifugation at 900×*g* for 30 min on 1.073 g/mL Percoll. The prepared cells were detached by treatment with 0.25% trypsin and transferred into culture flasks at a density of 1 × 10^4^ cells/cm^2^. The cells were evaluated for homogeneity through the detection of CD44 expression via cytometry, and seeded at 1 × 10^6^ cells/well into 12-well plates. After adherence for 24 h, bmMSCs were divided into control, model, and sample groups. In the control group, bmMSCs were incubated for 24 h in DMEM, while the cells from the model and sample groups were incubated in the presence of FeCl_2_ (100 μM) and then treated with by H_2_O_2_ (50 μM). The cells from the sample group were incubated with 3 μg/mL of sample.

After incubation for 10 min, the mixture of FeCl_2_ and H_2_O_2_ was removed. The cells were washed twice with 0.1 mol/L cold phosphate-buffered saline (PBS) and resuspended in a binding buffer at a concentration of 1 × 10^6^ cells/mL. About 100 μL of the solution (1 × 10^5^ cells) was transferred to a 5 mL culture tube and treated with 5 μL of fluorescein isothiocyanate (FITC)-Annexin V and 5 μL PI. The cells were vortexed and incubated for 15 min in the dark and treated with 400 μL of binding buffer, followed by analysis with flow cytometry (Accuri C6, Franklin Lakes, BD, USA) with standard software.

### Statistical analysis

Each experiment was performed in triplicates; the data were recorded as mean ± standard deviation (SD). The dose-response curves were plotted using Origin 2017 professional software (OriginLab, Northampton, MA, USA). The IC_50_ value was defined as the final concentration of 50% radical inhibition (or relative reducing power). Statistical comparisons were carried out with one-way analysis of variance (ANOVA) to detect significant differences using SPSS 13.0 software (SPSS Inc., Chicago, IL, USA) for Windows. A value of *p* < 0.05 was considered statistically significant.

## Results

### Preparation and HPLC analysis of LAEP

**Fig. 3:**
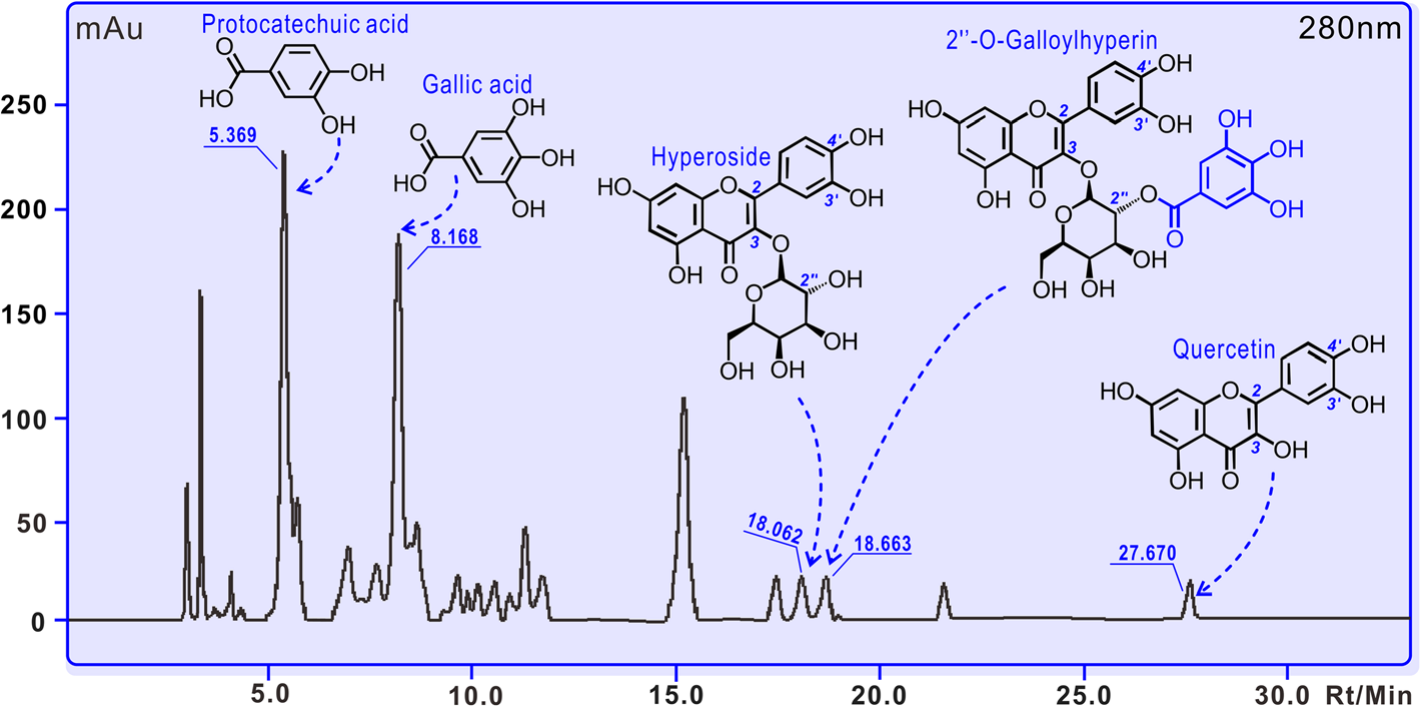
HPLC analysis of **LAEP**. **LAEP** means the lyophilized aqueous extract of *P. decorata*. The determining wavelength was 280 nm; the longitudinal axis was the strength of absorbance; mAu, milli-absorbance unit; Rt, retention time; Min, minute

The appearance of **LAEP** was a brown powder (Additional file 7), and its yield was calculated as 6.87%. The prepared **LAEP** was analyzed with HPLC and found to exhibit at least five different phenolic components, including protocatechuic acid, gallic acid, hyperoside, 2′′-*O*-galloylhyperin, and quercetin at 0.55, 0.31, 0.16, 0.12, and 0.08%, respectively. The retention times of these compounds were 5.369 min, 8.168 min, 18.062 min, 18.663 min, and 27.670 min, respectively (Fig. 3).

### Metal-reducing assays

In the study, two metal-reducing assays (i.e., FRAP assay and CUPRAC assay) were carried out to demonstrate the reducing power of antioxidants. As illustrated in Additional file 8, in FRAP assay, **LAEP** and its five phenolic components respectively increased the FRAP percentages in a dose-dependent manner. According to the IC_50_ values in Table 1, their Fe^3+^-reducing levels roughly increased in the order of protocatechuic acid < quercetin < gallic acid < hyperoside < 2“-*O*-galloylhyperin. In CUPRAC assay, **LAEP** and its five phenolic components respectively increased the CUPRAC percentages in a dose-dependent manner, and their order of Cu^2+^-reducing levels increased in the order of protocatechuic acid < gallic acid < quercetin < hyperoside < 2”-*O*-galloylhyperin (Table 1).

**Table 1:** The IC_50_ values of LAEP and its five phenolic components in various antioxidant assays

Assays	LAEP (μg/mL)	Protocatechuic acid (μM)	Gallic acid (μM)	Hyperoside (μM)	2′′-*O*-Galloylhyperin (μM)	Quercetin (μM)	Trolox (μM)
Fe^3+^-reducing	367.6 ± 11.2	105.5 ± 4.1^c^	54.3 ± 2.2^a^	51.4 ± 1.0^a^	48.9 ± 2.5^a^	59.3 ± 3.0^b^	97.3 ± 9.7
Cu^2+^-reducing	829.0 ± 13.9	183.7 ± 3.7^d^	148.3 ± 4.0^c^	105.6 ± 4.9^b^	71.9 ± 2.7^a^	123.5 ± 9.9^b^	373.9 ± 17.6
PTIO^•^-scavenging (pH 4.5)	131.7 ± 11.9	527.5 ± 6.6^b^	181.5 ± 5.6^a^	193.7 ± 28.4^a^	167.1 ± 14.7^a^	185.3 ± 15.3^a^	217.7 ± 5.0
PTIO^•^-scavenging (pH 7.4)	279.8 ± 58.3	437.0 ± 43.0^d^	179.7 ± 10.9^c^	135.2 ± 23.3^b^	92.2 ± 7.0^a^	103.6 ± 4.6^a^	142.9 ± 5.0
DPPH^•^-scavenging	82.4 ± 5.2	7.6 ± 0.5^c^	5.0 ± 0.2^b^	3.5 ± 0.1^b^	3.0 ± 0.1^a^	3.4 ± 0.2^a^	17.0 ± 1.5
ABTS^+•^-scavenging	42.3 ± 4.3	25.4 ± 0.4^d^	11.6 ± 0.4^c^	5.9 ± 0.2^b^	3.5 ± 0.1^a^	3.3 ± 0.1^a^	21.8 ± 0.6

The IC_50_ value was defined as the final concentration corresponding to 50% radical inhibition (or relative reducing power) obtained from the dose-response curves in Additional file 8, as analyzed by Origin 2017 professional software (OriginLab, Northampton, MA, USA). The IC_50_ values of the five phenolics with different superscripts (^a^, ^b^, ^c^, or ^d^) in the same row are significantly different (*p* < 0.05). Trolox ((±)-6-hydroxyl-2,5,7,8-tetramethlychromane-2-carboxylic acid) served as the positive control. **LAEP** means the lyophilized aqueous extract of *P. decorata*. Each experiment was performed in triplicates; the data were recorded as mean ± standard deviation (SD)

### Free radical-scavenging assays in vitro

As shown in Additional file 8, **LAEP** and its five phenolic components could successfully scavenge PTIO^•^ at pH 4.5 and pH 7.4. However, according to the IC_50_ values in Table 1, their relative PTIO^•^-scavenging levels at the same pH value were different from each other. Also, each of the five phenolic components exhibited different PTIO^•^-scavenging levels between pH 4.5 and pH 7.4.

The DPPH^•^-scavenging and ABTS^+•^-scavenging assays have been widely used in antioxidant studies. As seen in Additional file 8, **LAEP** and its five phenolic components concentration-dependently increased the DPPH^•^-scavenging and ABTS^+•^-scavenging percentages. The IC_50_ values were detailed in Table 1.

### Ultra-performance liquid chromatography coupled with electrospray ionization quadrupole time-of-flight tandem mass spectrometry (UPLC−ESI − Q − TOF − MS/MS) analysis

**Fig. 4:**
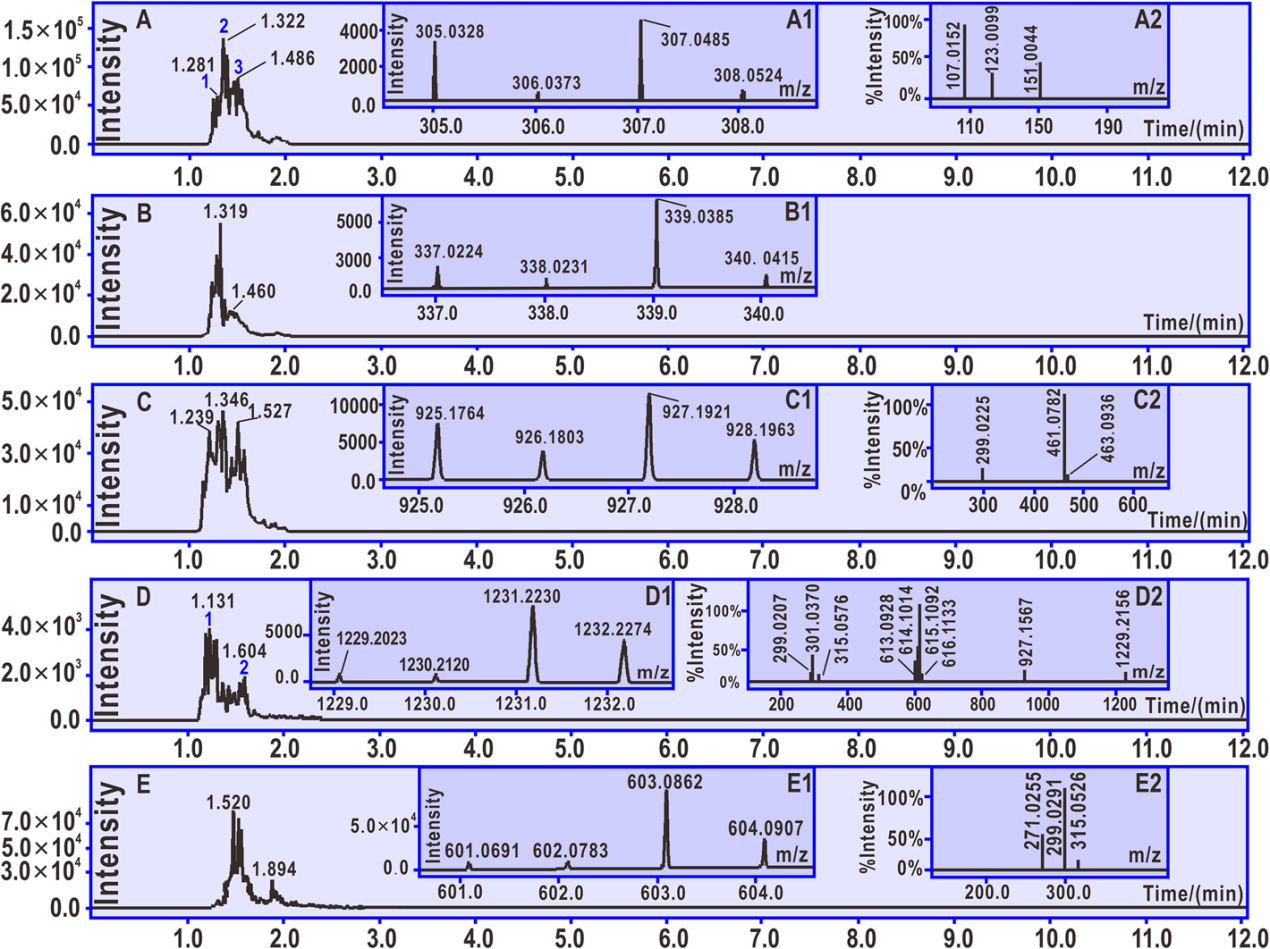
Results of UPLC-ESI-Q-TOF-MS/MS analysis. **a** Chromatogram of proto catechuic acid-protocatechuic acid dimer after the extraction of [C_14_H_10_O_8_-H]^−^.a1 Primary MS spectra of protocatechuic acid-protocatechuic acid dimer. a2 Secondary MS spectra of protocatechuic acid-protocatechuic acid dimer. **b** Chromatogram of gallic acid-gallic acid dimer after the extraction of [C_14_H_10_O_10_-H]^−^. (B1) Primary MS spectra of gallic acid-gallic acid dimer. **c** Chromatogram of hyperoside-hyperoside dimer after the extraction of [C_42_H_38_O_24_-H]^−^. c1 Primary MS spectra of hyperoside-hyperoside dimer. c2 Secondary MS spectra of hyperoside-hyperoside dimer. **d** Chromatogram of 2′′-O-galloylhyperin-2′′-O-galloylhyperin dimer after the extraction of [C_56_H_46_O_32_-H]^−^. d1 Primary MS spectra of 2′′-O-galloylhyperin-2′′-O-galloylhyperin dimer. d2 Secondary MS spectra of 2′′-O-galloylhyperin-2′′-O-galloylhyperin dimer. **e** Chromatogram of quercetin-quercetin dimer upon extraction of [C_30_H_18_O_14_-H]^−^. e1 Primary MS spectra of quercetin-quercetin dimer. e2 Secondary MS spectra of quercetin-quercetin dimer

Each of five phenolic components (i.e., protocatechuic acid, gallic acid, hyperoside, 2″-*O*-galloylhyperin, and quercetin) in **LAEP** was incubated with 4-methoxy-TEMPO^•^ for 24 h and analyzed with UPLC-ESI-Q-TOF-MS/MS. The chromatographic peaks, primary MS spectra (molecular ion peak), and secondary MS spectra were shown in Fig. 4.

### Protective effect on bmMSCs against ^•^OH-induced oxidative stress (flow cytometric assay)

**Fig. 5:**
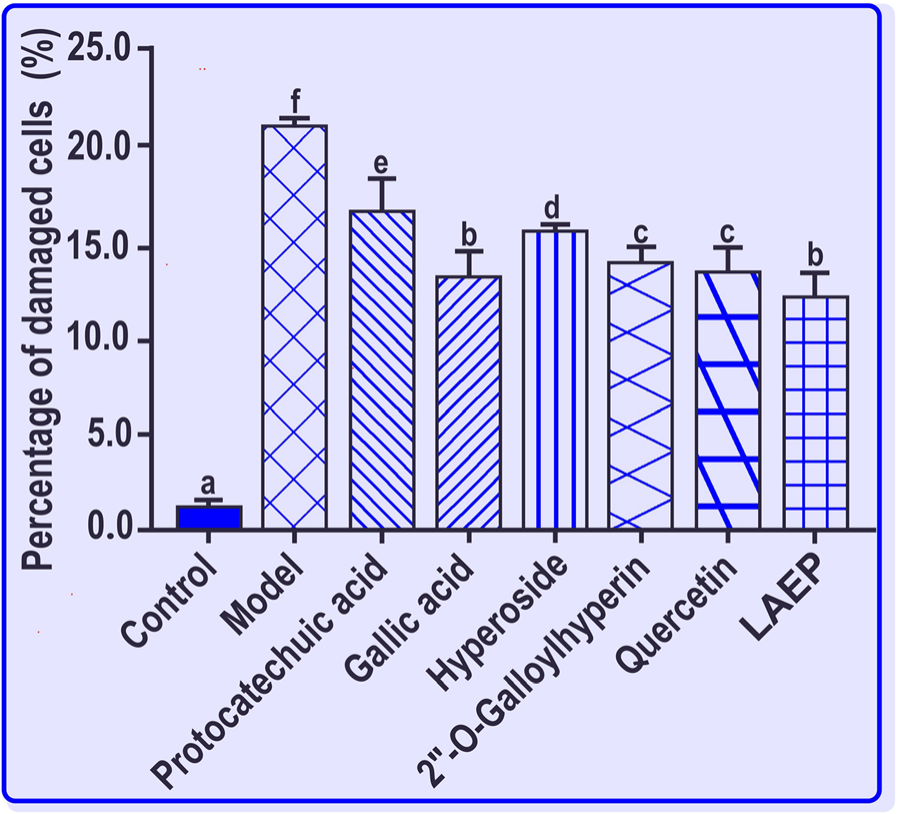
**LAEP** and its five phenolic components protect bmMSCs against Fenton-induced apoptosis. The percentage of damaged cells was assessed using a flow cytometric assay. Experiments were performed with three different batches of cells and each batch was tested in triplicates. Data are calculated as the mean ± SD values. **LAEP** means the lyophilized aqueous extract of *P. decorata*. The percentages of damaged cells with different superscripts (a, b, c, d, e, or f) are significantly different (*p* < 0.05)

As shown in Fig. 5, both **LAEP** and its five phenolic components had significantly (*p* < 0.05) decreased the percentages of damaged cells compared with the model group. In particular, the percentage of damaged cells was significantly (*p* < 0.05) lower in 2′′-*O*-galloylhyperin-treated group than in hyperoside-treated group.

## Discussion

In the study, five phenolic components have been found in *P. decorata*, including two phenolic acids (protocatechuic acid and gallic acid), and three flavonoid glucosides (hyperoside, 2′′-*O*-galloylhyperin, and quercetin) (Fig. 3). The detection of two phenolic acids undoubtedly has added new information of phytochemistry of *P. decorata* species [14, 15]. Regardless that the five phenolic components have also been expressed in *P. incarnata*; however, their contents in **LAEP** were slightly lower than those in *P. incarnata* [5, 32, 33]. The difference could be attributed to the species difference, the extraction processes employed. Furthermore, harvest seasons may also influence the contents of phenolics in *Pyrola* L. plants [33].

The five phenolic components and **LAEP** thus were further estimated using various antioxidant assays, such as FRAP and CUPRAC assays. The FRAP and CUPRAC assays are based on the electron transfer (ET) reaction at pH 3.6 and 7.4, respectively [34, 35]. Hence, the concentration-dependent increase of **LAEP** and its five phenolic components in reducing powers suggests that they could be involved in the ET pathway to exert antioxidant effects at pH 3.6 and 7.4.

To investigate whether the ET pathway could mediate radical-scavenging process, **LAEP** and its five phenolic components were further evaluated with PTIO^•^-scavenging assay. The PTIO^•^ radical is present as a stable form in aqueous buffers at various pH values, and its scavenging reaction at pH 4.5 was confirmed as a pure ET pathway by cyclic voltammetry [36]. The results demonstrated that **LAEP** and its five phenolic components could exhibit elevated PTIO^•^-scavenging activities at pH 4.5, indicative of their abilities to undergo ET reaction to scavenge radicals. However, at pH 7.4, **LAEP** and its five phenolic components could also effectively scavenge PTIO^•^ radical and their IC_50_ values at pH 7.4 were lower than those at pH 4.5 (Table 1). These pH-mediated effects suggest the involvement of the H^+^ transfer pathway in the PTIO^•^-scavenging process [27].

Besides aqueous buffers, aqueous solution was used as the experimental medium to evaluate the antioxidant properties in the ABTS^+•^-scavenging assay. Furthermore, an organic medium was used in the DPPH^•^-scavenging assay. The effectiveness of **LAEP** and its five phenolic components in the two assays revealed their ability to exert antioxidant actions in both organic and aqueous media.

**Fig. 6:**
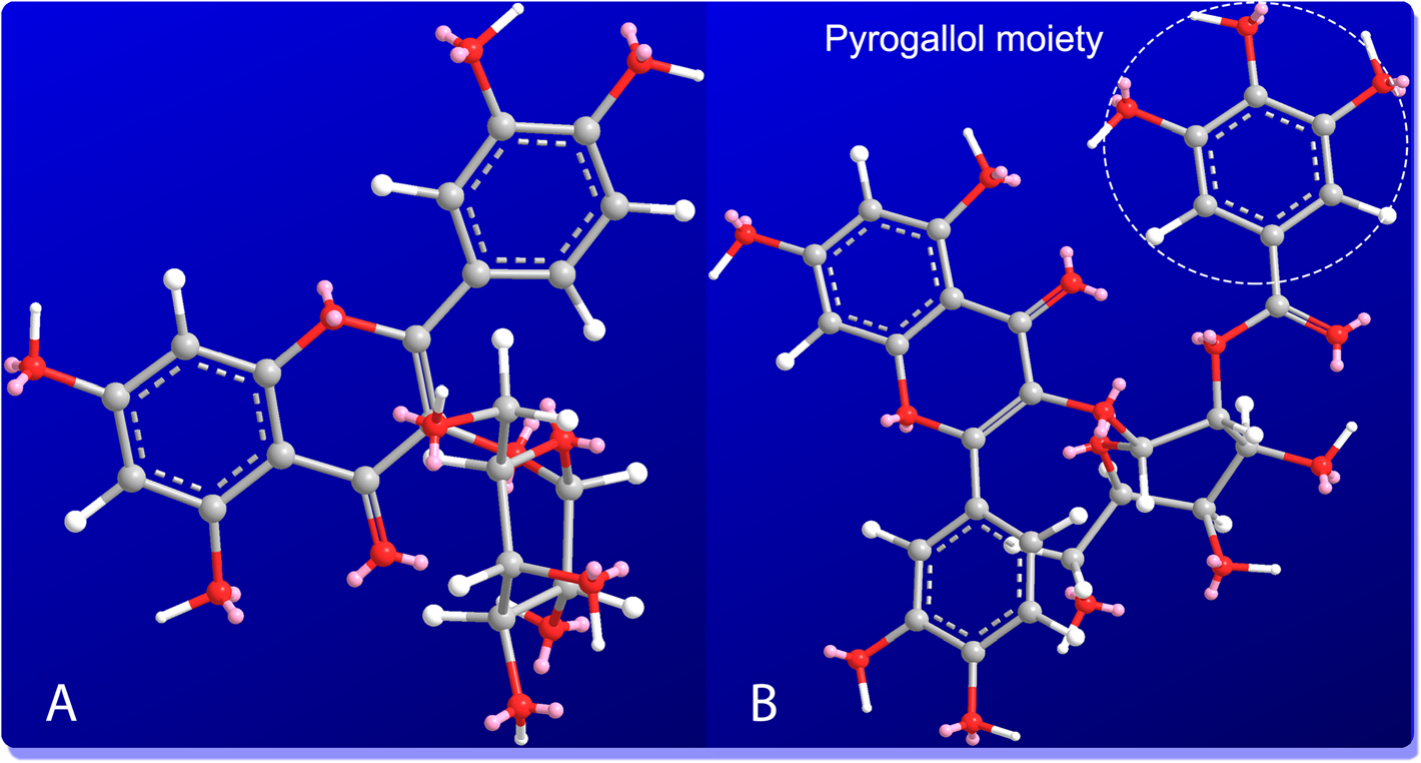
The ball-stick models of hyperoside (**a**) and 2′′-O-galloylhyperin (**b**) (red, O atom; white, H atom; gray, C atom; pink, long pair). The molecular models were created based on the calculation of molecular mechanics using Chem3D Pro 14.0 (CambridgeSoft, Cambridge, MA, USA)

The five detected phenolic components had different IC_50_ values in each antioxidant assay (Table 1). In particular, hyperoside showed higher IC_50_ values than 2′′-*O*-galloylhyperin in each of antioxidant assay, suggesting that 2′′-*O*-galloylhyperin had higher antioxidant effects based on ET and H^+^ transfer pathways than hyperoside. As previously discussed, the sole structural difference between hyperoside and 2′′-*O*-galloylhyperin is the presence of 2′′-*O*-galloyl moiety. Hence, the difference in their antioxidant activities could only be attributed to the role of galloylation. The pyrogallol moiety in 2′′-O-galloyl provides additional hydroxyl groups with radical-scavenging and metal-chelating abilities (Fig. 6) [24, 37–40].

Both ET and H^+^ transfer pathways are associated with the redox-based antioxidant mechanisms [41–43]. Through redox-based antioxidant mechanisms, new radical intermediates may be generated [30, 44]. These radical intermediates (including radical) may covalently link with each other to form a radical adduct. This is called as the radical adduct formation (RAF) mechanism. Dimerization however is one type of RAF. As seen in Fig. 4, protocatechuic acid, gallic acid, hyperoside, 2′′-*O*-galloylhyperin, and quercetin respectively produced the *m/z* values 306, 338, 926, 1230, and 602. These *m/z* values are exactly two units lower than twice their molecular weights of (M.W. 154, 170, 464, 616, and 302). We assumed that the two phenolic molecules were dimerized via one covalent bond. The observation of dimer formation indicates the presence of RAF mechanism in the antioxidant process associated with the five phenolic components in **LAEP**.

**Fig. 7:**
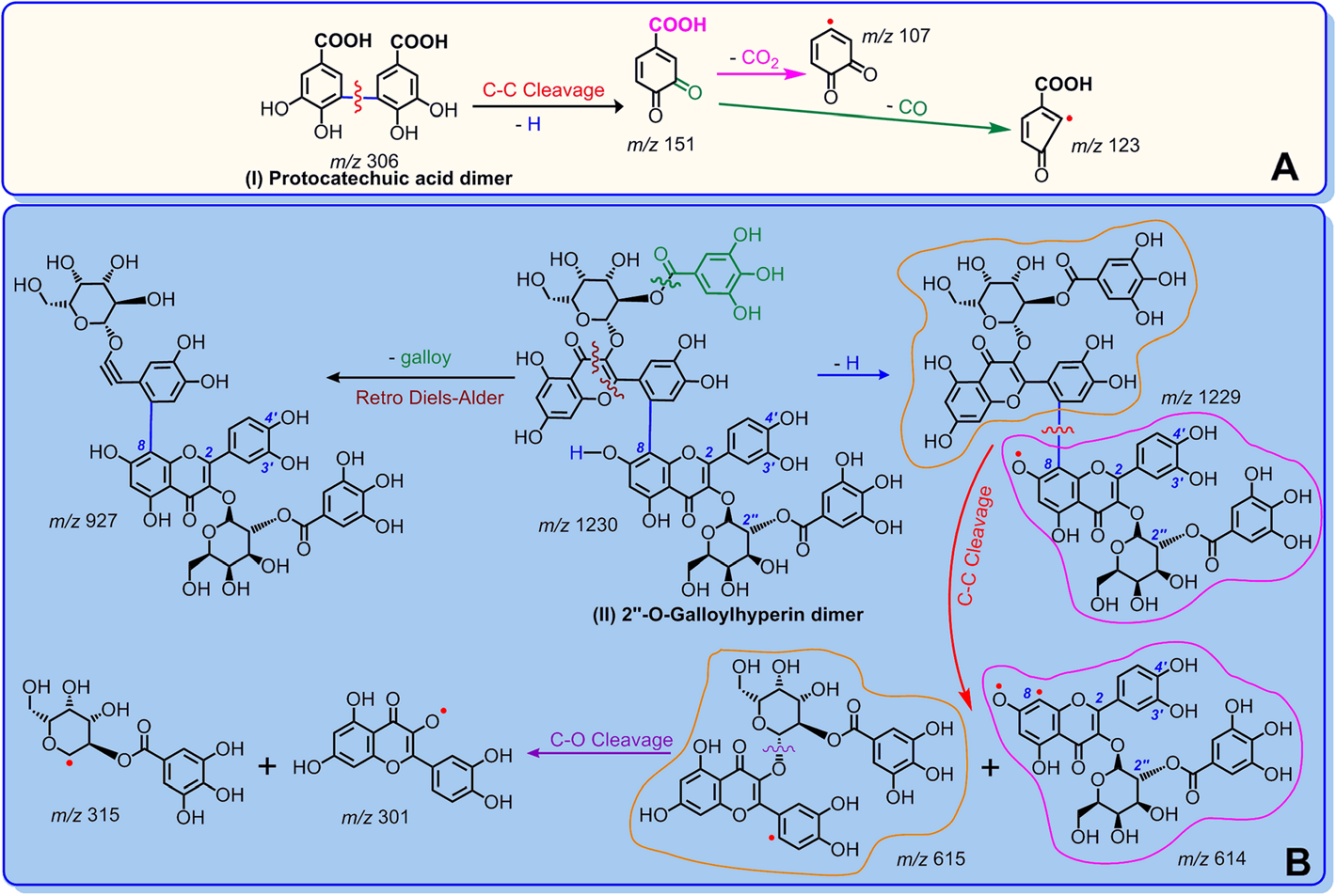
Proposed RAF products with MS elucidations of protocatechuic acid (**a**) and 2″-O-galloylhyperin (**b**) after mixed with 4-methoxy-TEMPO^•^. (The MS spectra were in the negative ion model, and the charge imposed by the MS field was not marked. Other linking sites between two phenolic moieties should not be excluded. Other reasonable cleavages should not be excluded in the MS elucidation)

Of these phenolic components, protocatechuic acid belongs to the phenolic acid family, whereas 2′′-*O*-galloylhyperin is a member of the flavonoid family. According to the MS/MS data, the dimeric products of protocatechuic acid and 2′′-*O*-galloylhyperin could be fully elucidated, as shown in Fig. 7. These elucidations further supported the presence of RAF mechanism. Furthermore, these elucidations clearly suggest that one consequence of RAF is the generation of phenolic-phenolic dimer. The dimer with covalent linkage, however, could effectively diminish the free radical to terminate the radical chain reaction [11].

The high resolution MS spectra provided an accurate *m/z* value for various fragments. For instance, the loss from *m/z* 151.0044 to 107.0152 was exactly the *m/z* value of CO_2_. The experimental *m/z* value of CO_2_ was 43.9892 (151.0044–107.0152) (Fig. 4a2 and Fig. 7a), while the calculated *m/z* value was 43.9899. The relative bias was only 1.6 × 10^− 5^. In addition, the *m/z* value from 151.0044 to 123.0099 was equal to 28. This loss was considered to be from N_2_ or CO. However, the accurate *m/z* value was 27.9945, indicating that the loss was related to CO (calculated *m/z* value 27.9949) than to N_2_ (calculated *m/z* value 28.0061) (Fig. 4A2 and Fig. 7a). The MS elucidations shown in Fig. 7 suggest the possible presence of other linking sites between two phenolic moieties, and that other reasonable cleavages should not be excluded in the MS elucidation.

To investigate whether **LAEP** and its five phenolic components could resist the oxidative stress-induced damage in biological system, we incubated bmMSCs damaged with Fenton reagent (an ^•^OH radical generator) in the presence of these compounds in an aqueous buffer. The percentage of damaged cells was characterized via flow cytometry*.* The results that **LAEP** and its five phenolic components could significantly (*p* < 0.05) decrease the percentages of damaged cells could be partly responsible for the pharmacological effect of *Luxiancao*. Furthermore, *P. decorata* and its phenolic components could function as therapeutic candidates for oxidative damages in bmMSC transplantation.

As mentioned above, the 2′′-*O*-galloylhyperin-treated group showed significantly (*p* < 0.05) higher cellular viability than hyperoside-treated group. Thus, 2′′-*O*-galloylhyperin could improve the cytoprotective effect of phenolic glycosides in aqueous buffer, consistent with the findings of antioxidant assays in organic and aqueous media. In bmMSC transplantation, galloylation of phenolic glycosides could be considered as a promising agent to improve the effectiveness of cytoprotectors.

## Conclusion

*P. decorata* contains at least five phenolic components, including protocatechuic acid, gallic acid, hyperoside, 2′′-*O*-galloylhyperin, and quercetin. These compounds may undergo redox-based pathways (such as ET and H^+^ transfer) and covalent-based pathway (i.e., RAF) to exhibit antioxidant activities. RAF could yield a stable phenolic-phenolic dimer. In comparison with hyperoside, 2′′-*O*-galloylhyperin showed better redox-based antioxidant activity (or cytoprotective activity) in both organic and aqueous media. The improvement stems from the 2′′-*O*-galloylation modification, which adds adjacent hydroxyl groups with radical-scavenging and metal-chelating abilities.

## Supplementary information

**Additional file 1.** Appearance and analysis certificate of protocatechuic acid.

**Additional file 2.** Appearance and analysis certificate of gallic acid.

**Additional file 3.** Appearance and analysis certificate of hyperoside.

**Additional file 4 **Appearance and analysis certificate of 2′′-*O*-galloylhyperin.

**Additional file 5.** Appearance and analysis certificate of quercetin.

**Additional file 6 **Originate plant and voucher specimen of *P. decorate.*

**Additional file 7 **Appearance of **LAEP**.

**Additional file 8.** Dose response curves and IC_50_ values.

## Data Availability

The datasets used during the current study are available from the corresponding author on reasonable request.

## Abbreviations

(NH_4_)_2_ABTS: 2,2′-azino-bis(3-ethylbenzo-thiazoline-6-sulfonic acid) diammonium salt
4-methoxy-TEMPO: 4-methoxy-2,2,6,6-tetramethylpiperidine-1-oxyl radical
ABTS^+•^: 2,2′-azino-bis(3-ethylbenzo-thiazoline-6-sulfonic acid) radical
bmMSCs: Bone marrow-derived mesenchymal stem cells
MSCs: Mesenchymal stem cells
CUPRAC: Cupric ions (Cu^2+^) reducing antioxidant capacity
DMEM: Dulbecco’s modified Eagle’s medium
DPPH: 1,1-diphenyl-2-picryl-hydrazl radical
ET: Electron transfer
FBS: Fetal bovine serum
FRAP: Ferric reducing antioxidant power
HAT: hydrogen atom transfer
LAEP: Lyophilized aqueous extract of *Pyrola decorata*
PCET: Proton-coupled electron transfer
PTIO: 2-phenyl-4,4,5,5-tetramethylimidazoline-1-oxyl 3-oxide radical
RAF: Radical adduct formation
ROS: Reactive oxygen species
SD: Standard deviation
SEPT: Sequential electron-proton transfer
SPLET: Sequential proton loss single-electron transfer
TCM: Traditional Chinese medicine
TPTZ: 2,4,6-tripyridyl triazine
Trolox: (±)-6-hydroxyl-2,5,7,8-tetramethlychromane-2-carboxylic acid
UPLC-ESI-Q-TOF-MS/MS: Ultra-performance liquid chromatography coupled with electrospray ionization quadrupole time-of-flight tandem mass spectrometry
