# Supplementary material for: Antioxidant and Cytoprotective effects of Pyrola decorata H. Andres and its five phenolic components
Source: BMC Complement Altern Med. 2019 Oct 21;19:275. doi: 10.1186/s12906-019-2698-y (PMC6805648; doi:10.1186/s12906-019-2698-y)
Supplement: Supplementary file 1 — Additional file 1. Appearance and analysis certificate of protocatechuic acid. [file 12906_2019_2698_MOESM1_ESM.pdf]

Addtional File 1: Appearance and analysis certificate of protocatechuic acid.

Antioxidant and Cytoprotective Effects of *Pyrola decorata* H. Andres and Its Five Phenolic Components

Ban Chen [1,2], Xican Li [1, 2,*], Jie Liu [3, 4], Wei Qin [3, 4], Minshi Liang [1, 2], Qianru Liu [1, 2], Dongfeng Chen [3, 4, *]

[1] School of Chinese Herbal Medicine, [2] Innovative Research & Development Laboratory of TCM, [3] School of Basic Medical Science, [4] The Research Center of Integrative Medicine, Guangzhou University of Chinese Medicine, Guangzhou, China, 510006.

[*] Corresponding author. **E-mail:** lixican@126.com; chen888@gzucm.edu.cn

E-mail Addresses

Ban Chen: imchenban@foxmail.com

Xican Li: lixican@126.com; lixc@gzucm.edu.cn

Jie Liu: 15014173165@163.com

Wei Qin: qinwei2017210@163.com

Minshi Liang: lminshi@outlook.com

Qianru Liu: liuqianru2333@163.com

Dongfeng Chen: chen888@gzucm.edu.cn

Address: School of Chinese Herbal Medicine, Guangzhou University of Chinese Medicine, Waihuan East Road No.232, Guangzhou Higher Education Mega Center, 510006, Guangzhou, China.

Homepage http://www.researchgate.net/profile/Xican_Li

Tel: +86-20-39358076

Fax: +86-20-38892690

Paper type: Research Article

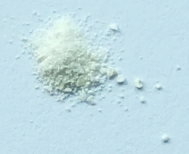

Protocatechuic acid CAS NO. 99-50-3

产品分析证书
Certificate of Analysis

中文名称：原儿茶酸

English Name：Protocatechuic Acid
别名**(Alias):** Carbohydroquinonic acid; Catechol-4-carboxylic acid; Hypogallic acid

产品编码**(Cat. No.)**:BP1155
CAS Number: 99-50-3
分子式**(M. F.)**: C7H6O4
分子量**(M. W.)**: 154.121

批号**(Batch No.)**: PRF8032441
报告日期**(Report date)**: 2017-03-24

Structure

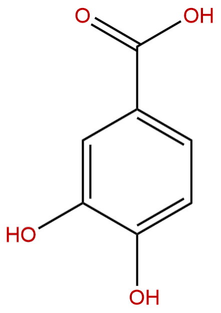

检验结果（**Analytical result**）：

检验项目（Test Item）	检验指标（Specifications）	检验结果（Results）
外观Appearance	Off-white powder	Off-white powder
干燥失重Loss on drying	＜3.0%	1.30%
纯度Purity (HPLC-DAD, 260nm)*	≥98.0%	99.79%
质谱Mass	154.121±1	Conforms
核磁NMR	Comply with the structure	Conforms

* 色谱图见附件（**Please find HPLC chromatography attached.**）

检测方法（**Test Method**）：**Column:** Thermo Hypersil GOLD AQ C18，4.6*250mm，5.0um; **Column temperature**: 30℃; **Detection Mode:** UV260nm; **Flow Rate:** 1.0ml/min; **Sample dissolution:** Methanol; **Mobile Phase: A**0.1% phosphoric acid **B,** Acetonitrile; **Gradient elution:** B, 2%-10%,15min.

贮存条件（**Storage**）:2~8℃, protected from light, keep package airproofed when not in use.

复测期（**Retest date**）:two years (2019-03-23) under conditions list above.

QC: Zhang Ling　　　　QA: Wu Qi

Date: 2017-03-24　　　　Date: 2017-03-24

备注**(Remarks)**: The sample solutions should be prepared and used on the same day. It is the best preparing the solutions immediately before use. If the solutons have to be made up in advance, it should be made as aliquots in tightly sealed vials at less than -20℃. Generally, these might be useable for up to two weeks.

In case of quality issue, please contact us within 15 days after receipt of the product.

Tel：+86-28-82633397　Fax：+86-28-82633165
http://www.phytopurify.com　Email：sales@biopurify.com　biopurify@gmail.com

SAMPLE INFORMATION

Sample Name:	Protocatechuic acid PRF8032441	Acquired By:	System
Sample Type:	Standard	Sample Set Name:	
Vial:	20	Acq. Method Set:	Protocatechuic acid
Injection #:	1	Processing Method:	Samples
Injection Volume:	5.00 ul	Channel Name:	260.0nm
Run Time:	25.0 Minutes	Proc. Chnl. Descr.:	PDA 260.0 nm
Date Acquired:	2017-3-24 10:38:48 CST		
Date Processed:	2017-3-24 11:24:19 CST		

Auto-Scaled Chromatogram

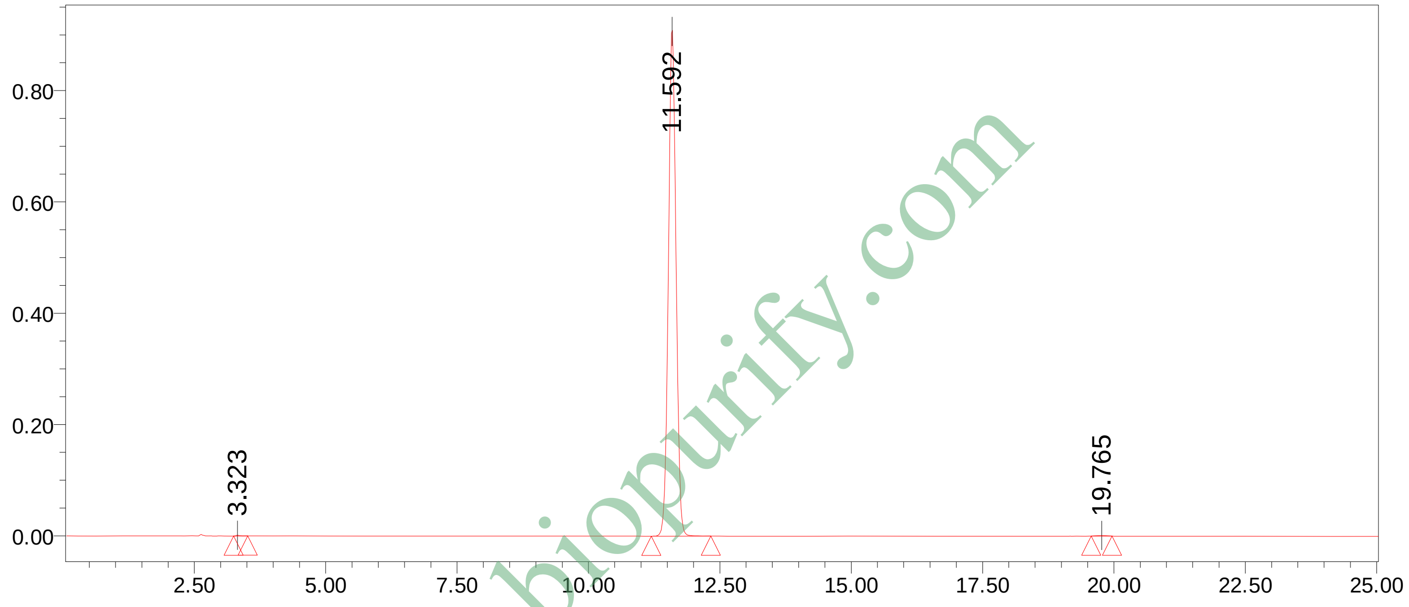

www.biopurify.com

Peak Results

	RT	Area	% Area	USP Plate Count	USP Resolution
1	3.323	7257	0.08	8837.28	
2	11.592	9239943	99.79	30136.00	39.45
3	19.765	12523	0.14	62075.74	27.61
