# Supplementary material for: Antioxidant and Cytoprotective effects of Pyrola decorata H. Andres and its five phenolic components
Source: BMC Complement Altern Med. 2019 Oct 21;19:275. doi: 10.1186/s12906-019-2698-y (PMC6805648; doi:10.1186/s12906-019-2698-y)
Supplement: Supplementary file 2 — Additional file 2. Appearance and analysis certificate of gallic acid. [file 12906_2019_2698_MOESM2_ESM.pdf]

Additional File 2: Appearance and analysis certificate of gallic acid.

Antioxidant and Cytoprotective Effects of *Pyrola decorata* H. Andres and Its Five Phenolic Components

Ban Chen [1,2], Xican Li [1, 2,*], Jie Liu [3, 4], Wei Qin [3, 4], Minshi Liang [1, 2], Qianru Liu [1, 2], Dongfeng Chen [3, 4, *]

[1] School of Chinese Herbal Medicine, [2] Innovative Research & Development Laboratory of TCM, [3] School of Basic Medical Science, [4] The Research Center of Integrative Medicine, Guangzhou University of Chinese Medicine, Guangzhou, China, 510006.

[*] Corresponding author. **E-mail:** lixican@126.com; chen888@gzucm.edu.cn

E-mail Addresses

Ban Chen: imchenban@foxmail.com

Xican Li: lixican@126.com; lixc@gzucm.edu.cn

Jie Liu: 15014173165@163.com

Wei Qin: qinwei2017210@163.com

Minshi Liang: lminshi@outlook.com

Qianru Liu: liuqianru2333@163.com

Dongfeng Chen: chen888@gzucm.edu.cn

Address: School of Chinese Herbal Medicine, Guangzhou University of Chinese Medicine, Waihuan East Road No.232, Guangzhou Higher Education Mega Center, 510006, Guangzhou, China.

Homepage http://www.researchgate.net/profile/Xican_Li

Tel: +86-20-39358076

Fax: +86-20-38892690

Paper type: Research Article

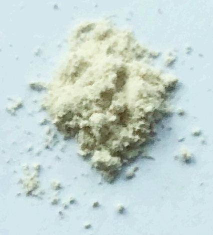

Gallic acid CAS NO. 149-91-7

成都普瑞法科技开发有限公司
Chengdu Biopurify Phytochemicals Ltd.

Add: No.11 Building, No. 388 Rongtaidadao CNSTP
Wenjiang Zone, Chengdu, Sichuan, 611130 China
TEL: 028-82633987 FAX: 028-82633165
E-mail: biopurify@gmail.com sales@biopurify.com
Web: www.phytopurify.com

Certificate of Analysis

Structure:

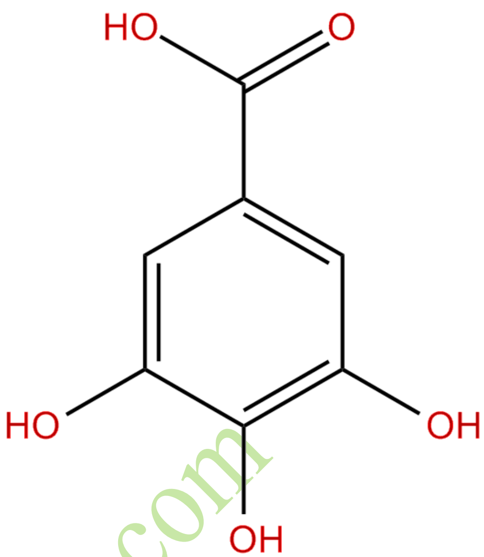

Product Name: Gallic acid

Other Name: 3,4,5-Trihydroxybenzoic acid

Catalogue No.: BP0608

Batch No.: 14051102

Report date: 2014-05-11

CAS Number: 149-91-7

Mol. Formula: $C_7H_6O_5$

Mol. Weight: 170.12

Type of compound:

Identification Method: Mass, NMR

Analysis Method of Purity: HPLC-DAD

www.biopurify.com

Analytical result:

Test	Specification	Results
Appearance	White powder	White powder
Loss on drying	＜3.0%	0.7%
Purity (HPLC, 270nm)*	≥98.0%	99.94%

* Please find HPLC chromatography attached.

Package: Brown vial or HDPE Plastic Bottle

Storage: Cool and Dry place, protected from light, keep package airproofed when not in use.

Expiration: two years (2016-05-11) under conditions list above.

QC: MengPan **QA:** Lianglei Zhang

Date: 2014-05-11 **Date:** 2014-05-11

Empower2 software Chengdu Biopurify Phytochemicals Ltd

SAMPLE INFORMATION

Sample Name:	Gallic acid14051102	Acquired By:	System
Sample Type:	Unknown	Sample Set Name:	
Vial:	1:A,1	Acq. Method Set:	Gallic acid
Injection #:	1	Processing Method:	QC
Injection Volume:	20.00 ul	Channel Name:	270.0nm
Run Time:	23.0 Minutes	Proc. Chnl. Descr.:	PDA 270.0 nm
Date Acquired:	2014-5-11 9:41:23 CST		
Date Processed:	2014-5-11 10:16:01 CST		

Auto-Scaled Chromatogram

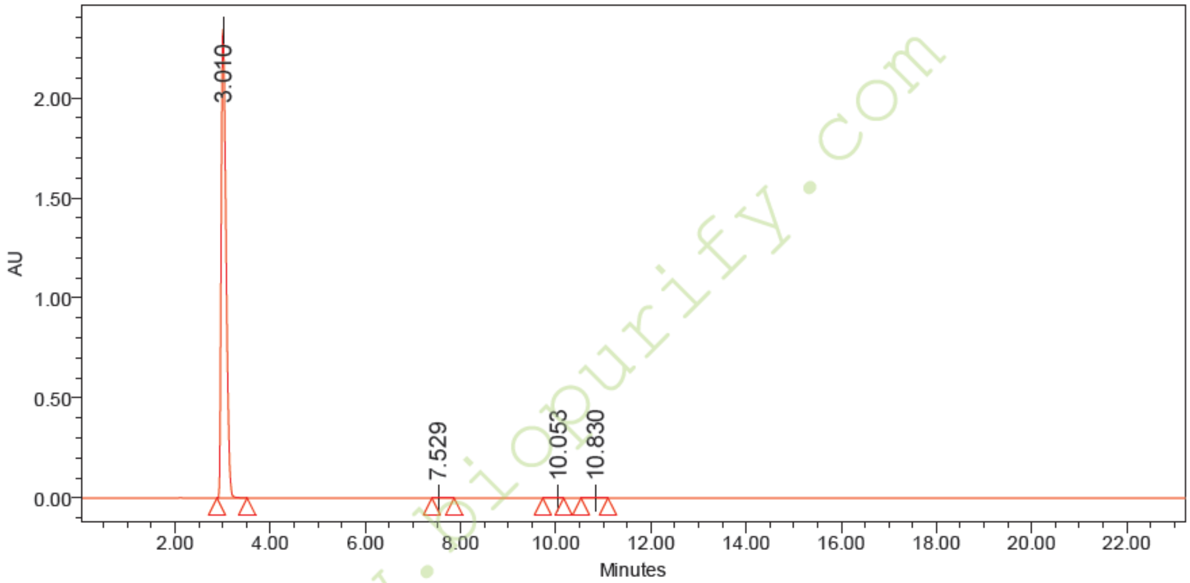

www.biopurify.com

Peak Results

	Name	RT	Area	% Area	USP Plate Count	USP Resolution
1		3.010	16328962	99.94	4026.03	
2		7.529	3545	0.02	32194.32	24.83
3		10.053	2423	0.01	12892.92	9.54
4		10.830	3763	0.02	27573.45	2.50
