# Supplementary material for: Antioxidant and Cytoprotective effects of Pyrola decorata H. Andres and its five phenolic components
Source: BMC Complement Altern Med. 2019 Oct 21;19:275. doi: 10.1186/s12906-019-2698-y (PMC6805648; doi:10.1186/s12906-019-2698-y)
Supplement: Supplementary file 3 — Additional file 3. Appearance and analysis certificate of hyperoside. [file 12906_2019_2698_MOESM3_ESM.pdf]

Additional File 3: Appearance and analysis certificate of hyperoside.

# Antioxidant and Cytoprotective Effects of *Pyrola decorata* H. Andres and Its Five Phenolic Components

Ban Chen [1,2], Xican Li [1, 2 *], Jie Liu [3, 4], Wei Qin [3, 4], Minshi Liang [1, 2], Qianru Liu [1, 2], Dongfeng Chen [3, 4, *]

[1] School of Chinese Herbal Medicine, [2] Innovative Research & Development Laboratory of TCM, [3] School of Basic Medical Science, [4] The Research Center of Integrative Medicine, Guangzhou University of Chinese Medicine, Guangzhou, China, 510006.

[*] Corresponding author. **E-mail:** lixican@126.com; chen888@gzucm.edu.cn

**E-mail Addresses**

Ban Chen: imchenban@foxmail.com

Xican Li: lixican@126.com; lixc@gzucm.edu.cn

Jie Liu: 15014173165@163.com

Wei Qin: qinwei2017210@163.com

Minshi Liang: lminshi@outlook.com

Qianru Liu: liuqianru2333@163.com

Dongfeng Chen: chen888@gzucm.edu.cn

**Address:** School of Chinese Herbal Medicine, Guangzhou University of Chinese Medicine, Waihuan East Road No.232, Guangzhou Higher Education Mega Center, 510006, Guangzhou, China.

**Homepage** http://www.researchgate.net/profile/Xican_Li

**Tel:** +86-20-39358076

**Fax:** +86-20-38892690

**Paper type:** Research Article

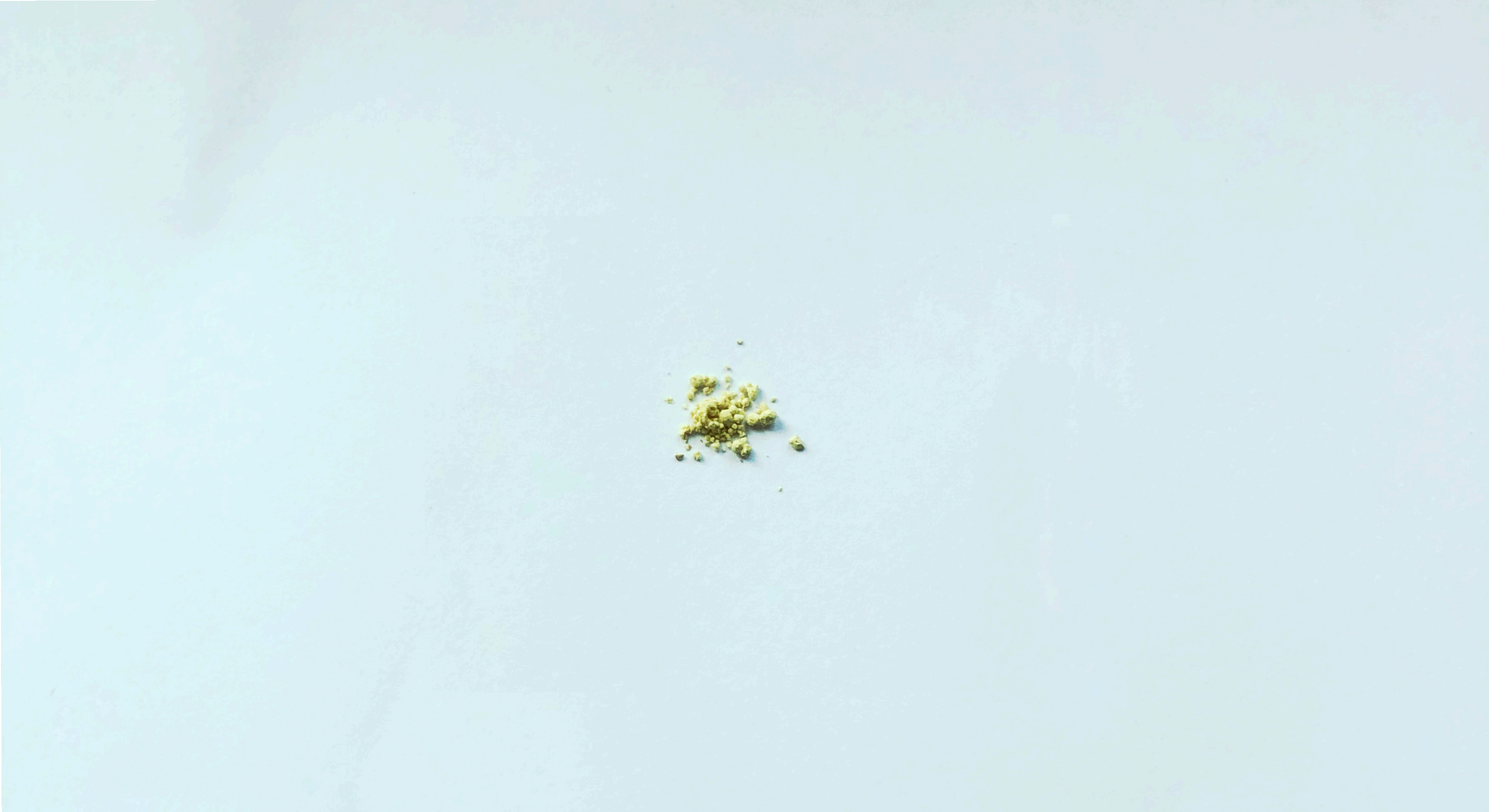

Hyperoside CAS NO. 482-36-0

成都普瑞法科技开发有限公司
Chengdu Biopurify Phytochemicals Ltd.

文件编码（DOC NO.）：**PRF-COA-BP075301**

# 产品分析证书
## Certificate of Analysis

**中文名称：金丝桃苷**

**English Name**：Hyperoside
**别名(Alias):** Quercetin 3-galactoside; Hyperin

**产品编码(Cat. No.):**BP0753
**CAS Number: 482-36-0**
**分子式(M. F.):** $C_{21}H_{20}O_{12}$
**分子量(M. W.)**: 464.379

**批号(Batch No.):** PRF7102522
**报告日期(Report date): 2016/10/25**

**Structure**

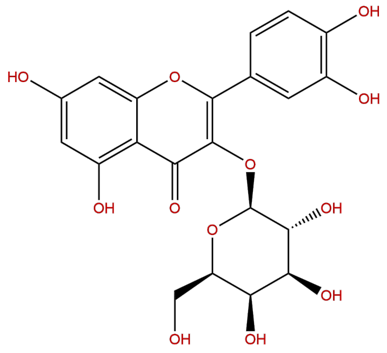


**检验结果（Analytical result）：**

| **检验项目（Test Item）** | **检验指标（Specifications）** | **检验结果（Results）** |
|---|---|---|
| 外观Appearance | Yellow powder | Yellow powder |
| 干燥失重Loss on drying | ＜3.0% | 1.28% |
| 纯度Purity (HPLC-DAD, 360nm)* | ≥98.0% | 99.13% |
| 质谱Mass | 464.379±1 | Conforms |
| 核磁NMR | Comply with the structure | Conforms |

*** 色谱图见附件（Please find HPLC chromatography attached.）**

贮存条件（Storage）:2~8℃
复测期（Retest date）:two years (2018-10-24) under conditions list above.
备注（Remarks）：如遇质量问题，请于收到产品之日起 15 日内与我们联系。
In case of quality issue, please contact us within 15 days after receipt of the product.

QC: Zhang Ling
Date: 2016年10月25日

Chengdu Biopurify Phytochemicals Ltd. 成都普瑞法科技开发有限公司 质检专用章 Quality Approval Seal

QA: Wu Qi
Date: 2016年10月25日

Tel：+86-28-82633987　　Fax：+86-28-82633165
http://www.biopurify.com　　Email：sales@biopurify.com　　biopurify@gmail.com

Chengdu Biopurify Phytochemicals Ltd.

# SAMPLE INFORMATION

| | | | |
|---|---|---|---|
| Sample Name: | Hyperoside PRF7102522 | Acquired By: | System |
| Sample Type: | Unknown | Sample Set Name: | |
| Vial: | 104 | Acq. Method Set: | Hyperoside |
| Injection #: | 1 | Processing Method: | Samples |
| Injection Volume: | 5.00 ul | Channel Name: | 360.0nm |
| Run Time: | 25.0 Minutes | Proc. Chnl. Descr.: | PDA 360.0 nm |
| Date Acquired: | 2016-10-25 12:13:29 CST | | |
| Date Processed: | 2016-10-25 12:44:17 CST | | |

## Auto-Scaled Chromatogram

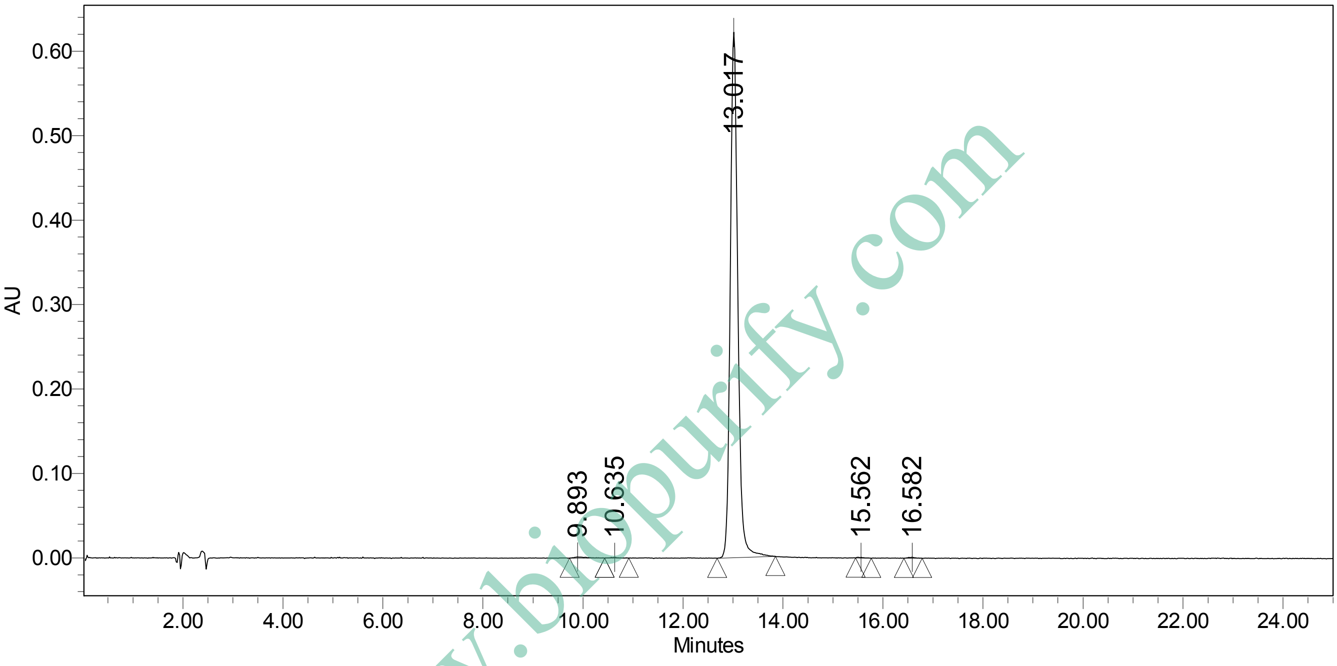


www.biopurify.com

## Peak Results

| | Name | RT | Area | % Area | USP Plate Count | USP Resolution |
|---|---|---|---|---|---|---|
| 1 | | 9.893 | 25408 | 0.39 | 3599.89 | |
| 2 | | 10.635 | 10727 | 0.16 | 17150.08 | 1.29 |
| 3 | | 13.017 | 6515083 | 99.13 | 37449.53 | 4.84 |
| 4 | | 15.562 | 7272 | 0.11 | 129837.82 | 8.63 |
| 5 | | 16.582 | 13701 | 0.21 | 39902.05 | 3.38 |

Reported by User: System
Report Method: Chengdu
Report Method ID: 23197


Project Name: 2016
Date Printed:
2016-10-25
13:06:26 PRC
