# Supplementary material for: Antioxidant and Cytoprotective effects of Pyrola decorata H. Andres and its five phenolic components
Source: BMC Complement Altern Med. 2019 Oct 21;19:275. doi: 10.1186/s12906-019-2698-y (PMC6805648; doi:10.1186/s12906-019-2698-y)
Supplement: Supplementary file 4 — Additional file 4 Appearance and analysis certificate of 2′′-O-galloylhyperin. [file 12906_2019_2698_MOESM4_ESM.pdf]

Additional File 4: Appearance and analysis certificate of 2''-O-galloylhyperin.

Antioxidant and Cytoprotective Effects of *Pyrola decorata* H. Andres and Its Five Phenolic Components

Ban Chen [1,2], Xican Li [1, 2,*], Jie Liu [3, 4], Wei Qin [3, 4], Minshi Liang [1, 2], Qianru Liu [1, 2], Dongfeng Chen [3, 4, *]

[1] School of Chinese Herbal Medicine, [2] Innovative Research & Development Laboratory of TCM, [3] School of Basic Medical Science, [4] The Research Center of Integrative Medicine, Guangzhou University of Chinese Medicine, Guangzhou, China, 510006.

[*] Corresponding author. **E-mail:** lixican@126.com; chen888@gzucm.edu.cn

E-mail Addresses

Ban Chen: imchenban@foxmail.com

Xican Li: lixican@126.com; lixc@gzucm.edu.cn

Jie Liu: 15014173165@163.com

Wei Qin: qinwei2017210@163.com

Minshi Liang: lminshi@outlook.com

Qianru Liu: liuqianru2333@163.com

Dongfeng Chen: chen888@gzucm.edu.cn

Address: School of Chinese Herbal Medicine, Guangzhou University of Chinese Medicine, Waihuan East Road No.232, Guangzhou Higher Education Mega Center, 510006, Guangzhou, China.

Homepage http://www.researchgate.net/profile/Xican_Li

Tel: +86-20-39358076

Fax: +86-20-38892690

Paper type: Research Article

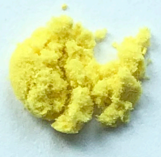

2’’-O-Galloylhyperin CAS NO. 53209-27-1

成都普瑞法科技开发有限公司
Chengdu Biopurify Phytochemicals Ltd.

文件编码（**DOC NO.**）：**PRF-COA-BP003601**

产品分析证书
Certificate of Analysis

中文名称：**2''-O-**没食子酰基金丝桃苷

English Name：2''-O-Galloylhyperin

别名 **(Alias):** Quercetin 3-beta-galactoside-2''-gallate

产品编码 **(Cat. No.):**BP0036

CAS Number: 53209-27-1

分子式 **(M. F.):** $C_{28}H_{24}O_{16}$

分子量 **(M. W.)**: 616.484

批号 **(Batch No.):** PRF7091808

报告日期 **(Report date): 2016/9/18**

Structure

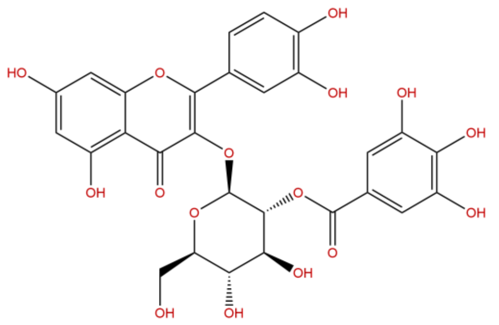

检验结果（**Analytical result**）:

检验项目（**Test Item**）	检验指标（**Specifications**）	检验结果（ **Results**）
外观Appearance	Yellow powder	Yellow powder
干燥失重Loss on drying	＜3.0%	1.34%
纯度Purity (HPLC-DAD, 265nm)*	≥98.0%	98.92%
质谱Mass	616.484±1	Conforms
核磁NMR	Comply with the structure	Conforms

*****色谱图见附件（**Please find HPLC chromatography attached.**）

贮存条件（Storage）:2~8℃

复测期（Retest date）:two years (2018-09-18) under conditions list above.

备注（Remarks）：如遇质量问题，请于收到产品之日起15日内与我们联系。

In case of quality issue, please contact us within 15 days after receipt of the product.

QC: Zhang Ling

Date:2016年9月18日

QA: Wu Qi

Date:2016年9月18日

Chengdu Biopurify Phytochemicals Ltd. 成都普瑞法科技开发有限公司 质检专用章 Quality Approval Seal

Tel：+86-28-82633987 Fax：+86-28-82633165

http://www.biopurify.com Email： sales@biopurify.com biopurify@gmail.com

Chengdu Biopurify Phytochemicals Ltd.

SAMPLE INFORMATION

Sample Name:	2 O Galloylhyperin PRF7091808	Acquired By:	System
Sample Type:	Unknown	Sample Set Name:	
Vial:	4	Acq. Method Set:	2 O Galloylhyperin
Injection #:	1	Processing Method:	Samples
Injection Volume:	10.00 ul	Channel Name:	265.0nm
Run Time:	25.0 Minutes	Proc. Chnl. Descr.:	PDA 265.0 nm
Date Acquired:	2016-9-18 16:19:07 CST		
Date Processed:	2016-9-18 16:47:48 CST		

Auto-Scaled Chromatogram

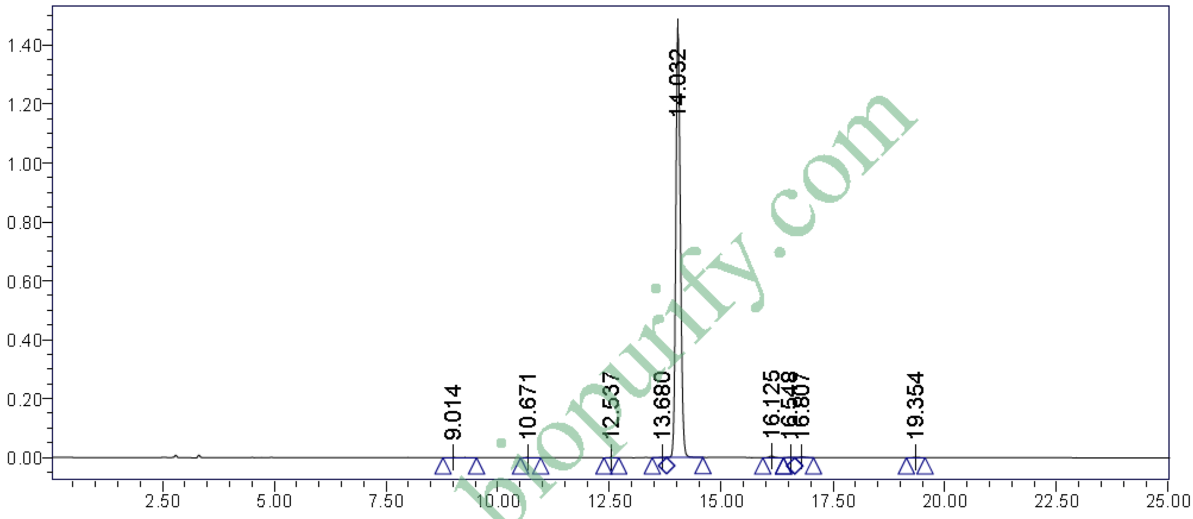

www.biopurify.com

Peak Results

	RT	Area	% Area	USP Plate Count	USP Resolution
1	9.014	6605	0.06	27079.58	
2	10.671	7145	0.07	36791.40	7.03
3	12.537	6395	0.06	59826.54	8.26
4	13.680	12617	0.12	57120.72	4.69
5	14.032	10501338	98.92	86939.83	1.51
6	16.125	36627	0.35	111139.92	10.72
7	16.548	5344	0.05	110053.17	2.09
8	16.807	27960	0.26	79099.39	1.15
9	19.354	11467	0.11	103744.35	10.38
