# Supplementary material for: Antioxidant and Cytoprotective effects of Pyrola decorata H. Andres and its five phenolic components
Source: BMC Complement Altern Med. 2019 Oct 21;19:275. doi: 10.1186/s12906-019-2698-y (PMC6805648; doi:10.1186/s12906-019-2698-y)
Supplement: Supplementary file 5 — Additional file 5. Appearance and analysis certificate of quercetin. [file 12906_2019_2698_MOESM5_ESM.pdf]

Additional File 5: Appearance and analysis certificate of quercetin.

Antioxidant and Cytoprotective Effects of *Pyrola decorata* H. Andres and Its Five Phenolic Components

Ban Chen [1,2], Xican Li [1, 2,*], Jie Liu [3, 4], Wei Qin [3, 4], Minshi Liang [1, 2], Qianru Liu [1, 2], Dongfeng Chen [3, 4, *]

[1] School of Chinese Herbal Medicine, [2] Innovative Research & Development Laboratory of TCM, [3] School of Basic Medical Science, [4] The Research Center of Integrative Medicine, Guangzhou University of Chinese Medicine, Guangzhou, China, 510006.

[*] Corresponding author. **E-mail:** lixican@126.com; chen888@gzucm.edu.cn

E-mail Addresses

Ban Chen: imchenban@foxmail.com

Xican Li: lixican@126.com; lixc@gzucm.edu.cn

Jie Liu: 15014173165@163.com

Wei Qin: qinwei2017210@163.com

Minshi Liang: lminshi@outlook.com

Qianru Liu: liuqianru2333@163.com

Dongfeng Chen: chen888@gzucm.edu.cn

Address: School of Chinese Herbal Medicine, Guangzhou University of Chinese Medicine, Waihuan East Road No.232, Guangzhou Higher Education Mega Center, 510006, Guangzhou, China.

Homepage http://www.researchgate.net/profile/Xican_Li

Tel: +86-20-39358076

Fax: +86-20-38892690

Paper type: Research Article

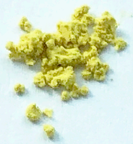

Quercetin CAS NO. 117-39-5

成都普瑞法科技开发有限公司
Chengdu Biopurify Phytochemicals Ltd.

文件编码（DOC NO.）：PRF-COA-BP118701

产品分析证书
Certificate of Analysis

中文名称：槲皮素

English Name：Quercetin
别名(Alias): Sophoretin; Meletin; Quercetol; Quertin; Ericin

产品编码(Cat. No.):BP1187
CAS Number: 117-39-5
分子式(M. F.): C15H10O7
分子量(M. W.): 302.238

批号(Batch No.): PRF8052241
报告日期(Report date): 2017-05-22

Structure

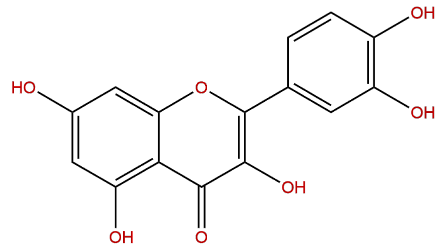

检验结果（Analytical result）：

检验项目（Test Item）	检验指标（Specifications）	检验结果（Results）
外观Appearance	Yellow powder	Yellow powder
干燥失重Loss on drying	＜3.0%	1.26%
纯度Purity (HPLC-DAD,370nm)*	≥98.0%	99.29%
质谱Mass	302.2±1	Conforms
核磁NMR	Comply with the structure	Conforms

* 色谱图见附件（**Please find HPLC chromatography attached.**）

检测方法（**Test Method**）：**Column:** Thermo Hypersil GOLD AQ C18，4.6*250mm，5.0um; **Column temperature**: 30℃; **Detection Mode:** UV370nm;**Flow Rate:** 1.0ml/min; **Sample dissolution:** Methanol; **Mobile Phase:** **A,**0.1% Phosphoric acid in water **B,** Acetonitrile;**Gradient elution:** B, 25%-40%,15min.

贮存条件（**Storage**）:2~8℃, protected from light, keep package airproofed when not in use.

复测期（**Retest date**）:two years (2019-05-21) under conditions list above.

QC: Zhang Ling
Date: 2017-05-22

QA: Wu Qi
Date: 2017-05-22

备注(Remarks): The sample solutions should be prepared and used on the same day. It is the best preparing the solutions immediately before use. If the solutons have to be made up in advance, it should be made as aliquots in tightly sealed vials at less than -20℃. Generally, these might be useable for up to two weeks.

In case of quality issue, please contact us within 15 days after receipt of the product.

Tel：+86-28-82633397 Fax：+86-28-82633165
http://www.phytopurify.com Email：sales@biopurify.com biopurify@gmail.com

SAMPLE INFORMATION

Sample Name:	Quercetin PRF8052241	Acquired By:	System
Sample Type:	Standard	Sample Set Name:	
Vial:	85	Acq. Method Set:	Quercetin
Injection #:	1	Processing Method:	Samples
Injection Volume:	10.00 ul	Channel Name:	370.0nm
Run Time:	25.0 Minutes	Proc. Chnl. Descr.:	PDA 370.0 nm
Date Acquired:	2017-5-22 12:12:18 CST		
Date Processed:	2017-5-22 12:51:47 CST		

Auto-Scaled Chromatogram

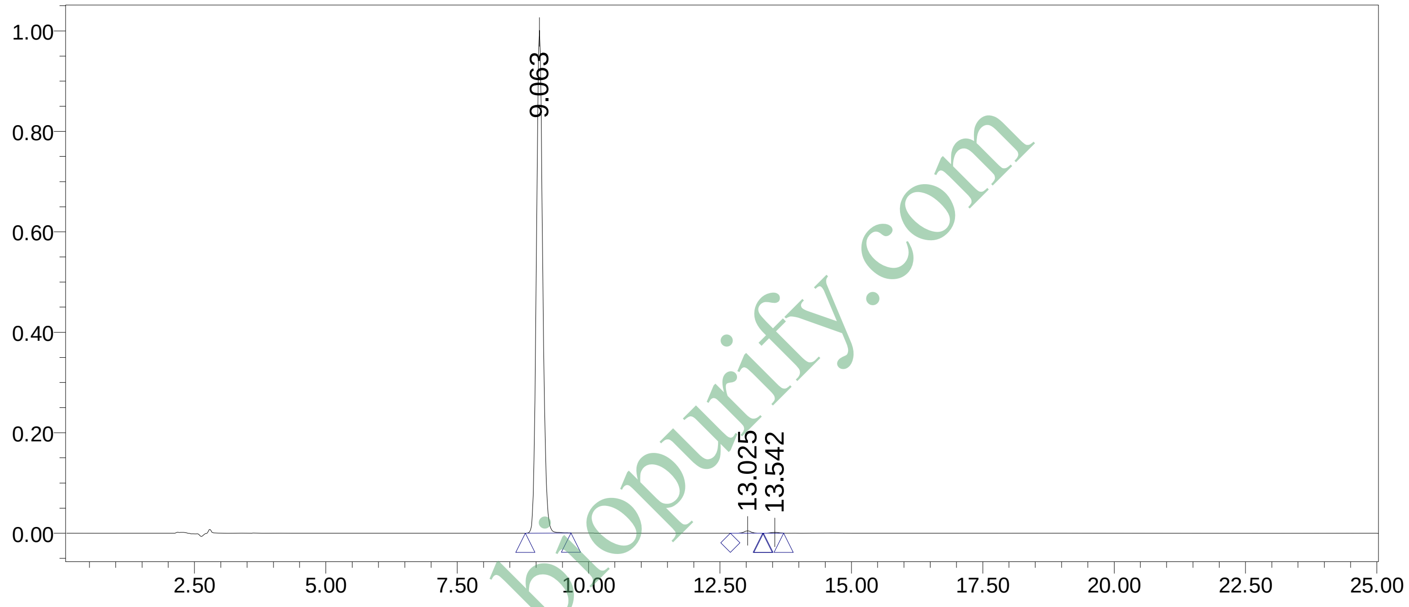

www.biopurify.com

Peak Results

	RT	Area	% Area	USP Plate Count	USP Resolution
1	9.063	8435567	99.29	26560.13	
2	13.025	46228	0.54	47875.99	16.78
3	13.542	14274	0.17	47434.70	2.11
