# Supplementary material for: Antioxidant and Cytoprotective effects of Pyrola decorata H. Andres and its five phenolic components
Source: BMC Complement Altern Med. 2019 Oct 21;19:275. doi: 10.1186/s12906-019-2698-y (PMC6805648; doi:10.1186/s12906-019-2698-y)
Supplement: Supplementary file 6 — Additional file 6 Originate plant and voucher specimen of P. decorate. [file 12906_2019_2698_MOESM6_ESM.docx]

Additional File 6: The originate plant and voucher specimen of *Pyrola decorata* H. Andres (*Luxiancao*).

**Antioxidant and Cytoprotective Effects of *Pyrola decorata* H. Andres and Its Five Phenolic Components**

Ban Chen ^1,2^, Xican Li ^1, 2*^, Jie Liu ^3, 4^, Wei Qin ^3, 4^, Minshi Liang ^1, 2^, Qianru Liu ^1, 2^, Dongfeng Chen ^3, 4, *^

^1^ School of Chinese Herbal Medicine, ^2^ Innovative Research & Development Laboratory of TCM, ^3^ School of Basic Medical Science, ^4^ The Research Center of Integrative Medicine, Guangzhou University of Chinese Medicine, Guangzhou, China, 510006.

^*^ Corresponding author. **E-mail:** [lixican@126.com](mailto:lixican@126.com); [chen888@gzucm.edu.cn](mailto:chen888@gzucm.edu.cn)

**E-mail Addresses**

Ban Chen**:** [imchenban@foxmail.com](mailto:imchenban@foxmail.com)

Xican Li**:** [lixican@126.com](mailto:lixican@126.com); [lixc@gzucm.edu.cn](mailto:lixc@gzucm.edu.cn)

Jie Liu**:** [15014173165@163.com](mailto:15014173165@163.com)

Wei Qin**:** [qinwei2017210@163.com](mailto:qinwei2017210@163.com)

Minshi Liang**:** [lminshi@outlook.com](mailto:lminshi@outlook.com)

Qianru Liu**:** [liuqianru2333@163.com](mailto:liuqianru2333@163.com)

Dongfeng Chen: [chen888@gzucm.edu.cn](mailto:chen888@gzucm.edu.cn)

**Address:** School of Chinese Herbal Medicine, Guangzhou University of Chinese Medicine, Waihuan East Road No.232, Guangzhou Higher Education Mega Center, 510006, Guangzhou, China.

**Homepage** <http://www.researchgate.net/profile/Xican_Li>

**Tel:** +86-20-39358076 **Fax:** +86-20-38892690 **Paper type:** Research Article


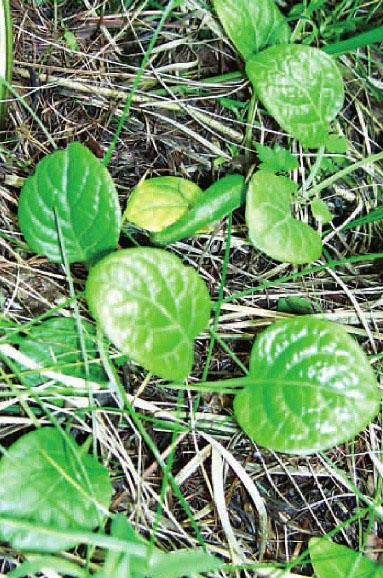


Fig. S6.1 The original plant of *Pyrola decorata* H. Andres


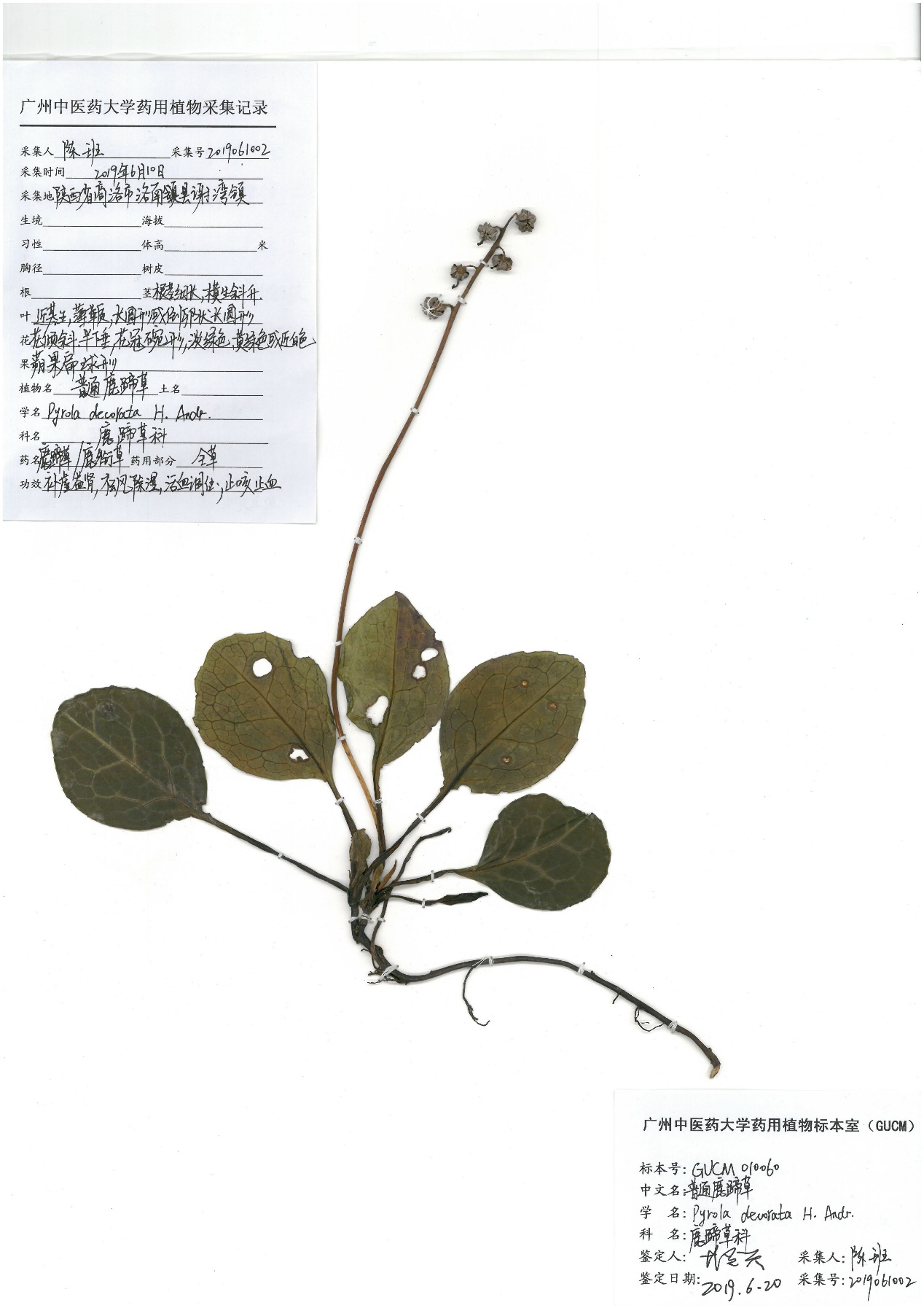


Fig. S6.2 The voucher specimen of *Pyrola decorata* H. Andres
