# Supplementary material for: Antioxidant and Cytoprotective effects of Pyrola decorata H. Andres and its five phenolic components
Source: BMC Complement Altern Med. 2019 Oct 21;19:275. doi: 10.1186/s12906-019-2698-y (PMC6805648; doi:10.1186/s12906-019-2698-y)
Supplement: Supplementary file 8 — Additional file 8. Dose response curves and IC50 values. [file 12906_2019_2698_MOESM8_ESM.doc]

Additional File 8: Dose response curves and IC_50_ values

**Antioxidant and Cytoprotective Effects of *Pyrola decorata* H. Andres and Its Five Phenolic Components**

Ban Chen ^1,2^, Xican Li ^1, 2,*^, Jie Liu ^3, 4^, Wei Qin ^3, 4^, Minshi Liang ^1, 2^, Qianru Liu ^1, 2^, Dongfeng Chen ^3, 4, *^

^1^ School of Chinese Herbal Medicine, ^2^ Innovative Research & Development Laboratory of TCM, ^3^ School of Basic Medical Science, ^4^ The Research Center of Integrative Medicine, Guangzhou University of Chinese Medicine, Guangzhou, China, 510006.

^*^ Corresponding author. **E-mail:** [lixican@126.com](mailto:lixican@126.com); [chen888@gzucm.edu.cn](mailto:chen888@gzucm.edu.cn)

^†^ These authors contributed equally to this work.

**E-mail Addresses**

Ban Chen**:** [imchenban@foxmail.com](mailto:imchenban@foxmail.com)

Xican Li**:** [lixican@126.com](mailto:lixican@126.com); [lixc@gzucm.edu.cn](mailto:lixc@gzucm.edu.cn)

Jie Liu**:** [15014173165@163.com](mailto:15014173165@163.com)

Wei Qin**:** [qinwei2017210@163.com](mailto:qinwei2017210@163.com)

Minshi Liang**:** [lminshi@outlook.com](mailto:lminshi@outlook.com)

Qianru Liu**:** [liuqianru2333@163.com](mailto:liuqianru2333@163.com)

Dongfeng Chen: [chen888@gzucm.edu.cn](mailto:chen888@gzucm.edu.cn)

**Address:** School of Chinese Herbal Medicine, Guangzhou University of Chinese Medicine, Waihuan East Road No.232, Guangzhou Higher Education Mega Center, 510006, Guangzhou, China.

**Homepage** <http://www.researchgate.net/profile/Xican_Li>

**Tel:** +86-20-39358076

**Fax:** +86-20-38892690

**Paper type:** Research Article

**Note:** This Supplementary File provides the original data of Table 1 in the main text. All data with underline are mentioned in main text (Table. 1).

1. FRAP Assay

Fig. S7.1 The does response curves of **LAEP**, the five phenolic components and positive control in FRAP assay.

Tab. S7.1 The comparison of IC_50_ values of **LAEP**, the five phenolic components and positive control in FRAP assay

|  | **LAEP** | Protocatechuic acid | Gallic acid | Hyperoside | 2"-O-Galloylhyperin | Quercetin | Trolox |
| --- | --- | --- | --- | --- | --- | --- | --- |
| μg/mL | 367.6±11.2 | 16.3±0.6 | 9.2±0.4 | 23.9±0.5 | 30.1±1.5 | 17.9±0.9 | 24.3±2.4 |
| μmol/L | N.D. | 105.5±4.1^c^ | 54.3±2.2^a^ | 51.4±1.0^a^ | 48.9±2.5^a^ | 59.3±3.0^b^ | 97.3±9.7^c^ |

The IC_50_ value was defined as the final concentration of 50% radical inhibition (or relative reducing power), they were obtained from the dose-response curves in Fig. S7.1, which were analyzed by Origin 2017 professional software (OriginLab, Northampton, MA, USA). Different letters (a, b, or c) in the same row are significantly (p < 0.05) different among phenolic components and the positive control Trolox, they were analyzed by one-way ANOVA to detect significant differences using SPSS 13.0 software (SPSS Inc., Chicago, IL) for windows. Each experiment was performed in triplicate; the data were recorded as mean ± SD (standard deviation).

2. CUPRAC Assay

Fig. S7.2 The does response curves of **LAEP**, the five phenolic components and positive control in CUPRAC assay.

Tab. S7.2 The comparison of IC_50_ values of **LAEP**, the five phenolic components and positive control in CUPRAC assay

|  | **LAEP** | Protocatechuic acid | Gallic acid | Hyperoside | 2"-O-Galloylhyperin | Quercetin | Trolox |
| --- | --- | --- | --- | --- | --- | --- | --- |
| μg/mL | 829.0±13.9 | 28.3±0.6 | 25.2±0.7 | 49.0±2.3 | 44.1±1.7 | 37.3±3.0 | 93.6±4.4 |
| μmol/L | N.D. | 183.7±3.7^d^ | 148.3±4.0^c^ | 105.6±4.9^b^ | 71.9±2.7^a^ | 123.5±9.9^b^ | 373.9±17.6^e^ |

The IC_50_ value was defined as the final concentration of 50% radical inhibition (or relative reducing power), they were obtained from the dose-response curves in Fig. S7.2, which were analyzed by Origin 2017 professional software (OriginLab, Northampton, MA, USA). Different letters (a, b, c, d, or e) in the same row are significantly (p < 0.05) different among phenolic components and the positive control Trolox, they were analyzed by one-way ANOVA to detect significant differences using SPSS 13.0 software (SPSS Inc., Chicago, IL) for windows. Each experiment was performed in triplicate; the data were recorded as mean ± SD (standard deviation).

3. PTIO^•^-Scavenging Assay (pH 4.5)

Fig. S7.3 The does response curves of **LAEP**, the five phenolic components and positive control in PTIO^•^-scavenging assay (pH 4.5).

Tab. S7.3 The comparison of IC_50_ values of **LAEP**, the five phenolic components and positive control in PTIO^•^-scavenging assay (pH 4.5)

|  | **LAEP** | Protocatechuic acid | Gallic acid | Hyperoside | 2"-O-Galloylhyperin | Quercetin | Trolox |
| --- | --- | --- | --- | --- | --- | --- | --- |
| μg/mL | 131.7±11.9 | 81.2±1.0 | 30.9±1.0 | 89.9±13.2 | 102.9±9.1 | 56.0±4.6 | 54.4±1.2 |
| μmol/L | N.D. | 527.5±6.6^c^ | 181.5±5.6^a^ | 193.7±28.4^a^ | 167.1±14.7^a^ | 185.3±15.3^a^ | 217.7±5.0^b^ |

The IC_50_ value was defined as the final concentration of 50% radical inhibition (or relative reducing power), they were obtained from the dose-response curves in Fig. S7.3, which were analyzed by Origin 2017 professional software (OriginLab, Northampton, MA, USA). Different letters (a, b, or c) in the same row are significantly (p < 0.05) different among phenolic components and the positive control Trolox, they were analyzed by one-way ANOVA to detect significant differences using SPSS 13.0 software (SPSS Inc., Chicago, IL) for windows. Each experiment was performed in triplicate; the data were recorded as mean ± SD (standard deviation).

4. PTIO^•^-Scavenging Assay (pH 7.4)

Fig. S7.4 The does response curves of **LAEP**, the five phenolic components and positive control in PTIO^•^-scavenging assay (pH 7.4).

Tab. S7.4 The comparison of IC_50_ values of **LAEP**, the five phenolic components and positive control in PTIO^•^-scavenging assay (pH 7.4)

|  | **LAEP** | Protocatechuic acid | Gallic acid | Hyperoside | 2"-O-Galloylhyperin | Quercetin | Trolox |
| --- | --- | --- | --- | --- | --- | --- | --- |
| μg/mL | 279.8±58.3 | 67.3±6.6 | 30.9±1.9 | 62.7±10.8 | 56.8±4.3 | 31.3±1.4 | 35.7±1.2 |
| μmol/L | N.D. | 437.0±43.0^d^ | 179.7±10.9^c^ | 135.2±23.3^b^ | 92.2±7.0^a^ | 103.6±4.6^a^ | 142.9±5.0^b^ |

The IC_50_ value was defined as the final concentration of 50% radical inhibition (or relative reducing power), they were obtained from the dose-response curves in Fig. S7.4, which were analyzed by Origin 2017 professional software (OriginLab, Northampton, MA, USA). Different letters (a, b, c, or d) in the same row are significantly (p < 0.05) different among phenolic components and the positive control Trolox, they were analyzed by one-way ANOVA to detect significant differences using SPSS 13.0 software (SPSS Inc., Chicago, IL) for windows. Each experiment was performed in triplicate; the data were recorded as mean ± SD (standard deviation).

5. DPPH^•^-Scavenging Assay

Fig. S7.5 The does response curves of **LAEP**, the five phenolic components and positive control in DPPH^•^-scavenging assay.

Tab. S7.5 The comparison of IC_50_ values of **LAEP**, the five phenolic components and positive control in DPPH^•^-scavenging assay

|  | **LAEP** | Protocatechuic acid | Gallic acid | Hyperoside | 2"-O-Galloylhyperin | Quercetin | Trolox |
| --- | --- | --- | --- | --- | --- | --- | --- |
| μg/mL | 82.4±5.2 | 1.2±0.1 | 0.9±0.1 | 1.6±0.1 | 1.8±0.1 | 1.0±0.1 | 4.3±0.4 |
| μmol/L | N.D. | 7.6±0.5^c^ | 5.0±0.2^b^ | 3.5±0.1^b^ | 3.0±0.1^a^ | 3.4±0.2^a^ | 17.0±1.5^d^ |

The IC_50_ value was defined as the final concentration of 50% radical inhibition (or relative reducing power), they were obtained from the dose-response curves in Fig. S7.5, which were analyzed by Origin 2017 professional software (OriginLab, Northampton, MA, USA). Different letters (a, b, c, or d) in the same row are significantly (p < 0.05) different among phenolic components and the positive control Trolox, they were analyzed by one-way ANOVA to detect significant differences using SPSS 13.0 software (SPSS Inc., Chicago, IL) for windows. Each experiment was performed in triplicate; the data were recorded as mean ± SD (standard deviation).

6. ABTS^+•^-Scavenging Assay

Fig. S7.6 The does response curves of **LAEP**, the five phenolic components and positive control in ABTS^+•^-scavenging assay.

Tab. S7.6 The comparison of IC_50_ values of **LAEP**, the five phenolic components and positive control in ABTS^+•^-scavenging assay

|  | **LAEP** | Protocatechuic acid | Gallic acid | Hyperoside | 2"-O-Galloylhyperin | Quercetin | Trolox |
| --- | --- | --- | --- | --- | --- | --- | --- |
| μg/mL | 42.3±4.3 | 4.0±0.1 | 2.0±0.1 | 2.7±0.1 | 2.2±0.1 | 1.0±0.1 | 5.4±0.1 |
| μmol/L | N.D. | 25.4±0.4^d^ | 11.6±0.4^c^ | 5.9±0.2^a^ | 3.5±0.1^a^ | 3.3±0.1^a^ | 21.8±0.6^b^ |

The IC_50_ value was defined as the final concentration of 50% radical inhibition (or relative reducing power), they were obtained from the dose-response curves in Fig. S7.6, which were analyzed by Origin 2017 professional software (OriginLab, Northampton, MA, USA). Different letters (a, b, c, or d) in the same row are significantly (p < 0.05) different among phenolic components and the positive control Trolox, they were analyzed by one-way ANOVA to detect significant differences using SPSS 13.0 software (SPSS Inc., Chicago, IL) for windows. Each experiment was performed in triplicate; the data were recorded as mean ± SD (standard deviation).
